# Whole-genome sequencing of two *Streptomyces* strains isolated from the sand dunes of Sahara

**DOI:** 10.1186/s12864-021-07866-x

**Published:** 2021-07-27

**Authors:** Chahira Zerouki, Farid Bensalah, Suvi Kuittinen, Ari Pappinen, Ossi Turunen

**Affiliations:** 1 School of Forest Sciences, University of Eastern Finland, FI-80101 Joensuu, Finland; 2 Laboratory of Microbial Genetics, Department of Biology, University ORAN 1, 31000 Oran, Algeria

**Keywords:** Sahara, Soil, *Streptomyces*, Complete genome sequence, Secondary metabolites, Enzymes

## Abstract

**Background:**

Sahara is one of the largest deserts in the world. The harsh climatic conditions, especially high temperature and aridity lead to unique adaptation of organisms, which could be a potential source of new metabolites. In this respect, two Saharan soils from El Oued Souf and Beni Abbes in Algeria were collected. The bacterial isolates were selected by screening for antibacterial, antifungal, and enzymatic activities. The whole genomes of the two native Saharan strains were sequenced to study desert *Streptomyces* microbiology and ecology from a genomic perspective.

**Results:**

Strains Babs14 (from Beni Abbes, Algeria) and Osf17 (from El Oued Souf, Algeria) were initially identified by 16S rRNA sequencing as belonging to the *Streptomyces* genus. The whole genome sequencing of the two strains was performed using Pacific Biosciences Sequel II technology (PacBio), which showed that Babs14 and Osf17 have a linear chromosome of 8.00 Mb and 7.97 Mb, respectively. The number of identified protein coding genes was 6910 in Babs14 and 6894 in Osf17. No plasmids were found in Babs14, whereas three plasmids were detected in Osf17. Although the strains have different phenotypes and are from different regions, they showed very high similarities at the DNA level. The two strains are more similar to each other than either is to the closest database strain. The search for potential secondary metabolites was performed using antiSMASH and predicted 29 biosynthetic gene clusters (BGCs). Several BGCs and proteins were related to the biosynthesis of factors needed in response to environmental stress in temperature, UV light and osmolarity.

**Conclusion:**

The genome sequencing of Saharan *Streptomyces* strains revealed factors that are related to their adaptation to an extreme environment and stress conditions. The genome information provides tools to study ecological adaptation in a desert environment and to explore the bioactive compounds of these microorganisms. The two whole genome sequences are among the first to be sequenced for the *Streptomyces* genus of Algerian Sahara. The present research was undertaken as a first step to more profoundly explore the desert microbiome.

**Supplementary Information:**

The online version contains supplementary material available at 10.1186/s12864-021-07866-x.

## Background

*Streptomyces* belongs to the Actinobacteria phylum and is one of the most diverse groups, primarily found in soil and aquatic habitats. *Streptomyces* bacteria are filamentous, sporulating, gram-positive and metabolize a broad range of carbon sources. Furthermore, *Streptomyces* encompasses the biosynthesis of several secondary metabolites with industrial implications [1–4]. Most of the compounds of microbial origin discovered to date with antibiotic, antitumor, or immunosuppressive activities are derived from *Streptomyces*. It has been suggested that these bacteria might produce much more metabolites than have been identified to date [5]. *Streptomyces* bacteria are also valuable from an environmental and ecological standpoint. These bacteria are considered as key players in the decomposition of biomass, especially due to their capability to degrade diverse organic compounds.

Currently, the world is facing the harmful emergence of multidrug-resistant pathogens. This necessitates a search for metabolically potent species that could be a source of new secondary metabolites. In nature, bacterial secondary metabolites play important ecological and physiological roles. Their contribution is more topical under extreme conditions, where the bacteria have evolved strategies to survive and proliferate under adverse circumstances [6, 7]. Many organisms in extreme environments may represent new taxa and could provide a valuable resource for new bioactive compounds and enzymes for industrial applications [8–10].

The Algerian Saharan soil is a unique ecosystem. Temperatures typically vary between − 5 and + 45 °C. The Sahara is a very challenging environment for microorganisms, especially due to low humidity levels, high ultraviolet (UV) radiation, presence of inorganic oxidants, starvation, and the physical instability caused by strong winds [6]. Although, plant-free desert ecosystems represent lowered variation capacity [11], the Saharan soils under extreme climate conditions still represent ecosystems with significant biodiversity [12]. Previous investigations have demonstrated an abundance of actinomycetes in Saharan soils [10], and in arid Atacama Desert soils in Chile [13, 14]. Novel bioactive molecules [15] and novel species of *Actinopolyspora* [16–18], halophilic actinobacteria and *Actinomadura* [19–21] have been previously isolated from the Algerian Sahara.

The present study describes the isolation and characterization of *Streptomyces* strains from south-west Algeria (Beni Abbes) and south-east Algeria (El Oued Souf), and their screening for antimicrobial, antifungal, and enzymatic activities. In addition, whole genome sequencing and bioinformatic tools were used to analyse the genomic DNA and protein sequences of these two native Saharan strains.

## Results

### Strain isolation and selection

**Fig. 1:**
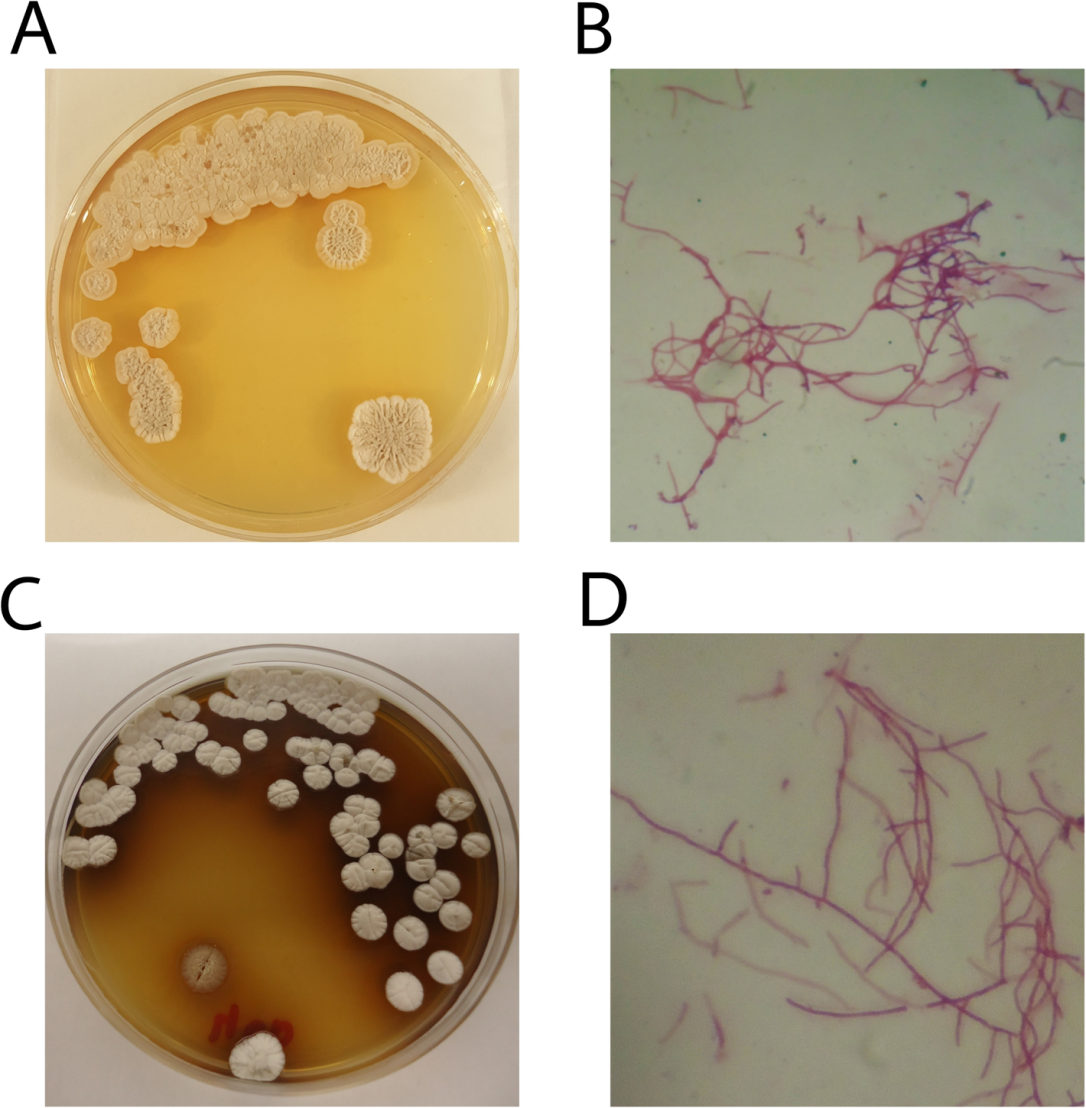
Morphological characteristics of strains Babs14 (**A**, **B**) and Osf17 (**C**, **D**). Macroscopic morphology on ISP2 agar (pH 7) after incubation for 7 days (**A**, **C**), and microscopic filamentous morphology of the mycelium under light microscopy (magnification 10 × 100) stained with Fuchsin (**C**, **D**)

A total of 12 isolates that exhibited filamentous features were identified, which may indicate Actinobacteria. Two strains from different samples were the subject of our study: Babs14 from Beni Abbes and Osf17 from El Oued Souf (Fig. 1). The isolate Babs14 was cultivated and purified on ISP2 after 4 days of incubation, and Osf17 was isolated from SCA medium after 3 days of incubation at 30 °C.

**Fig. 2:**
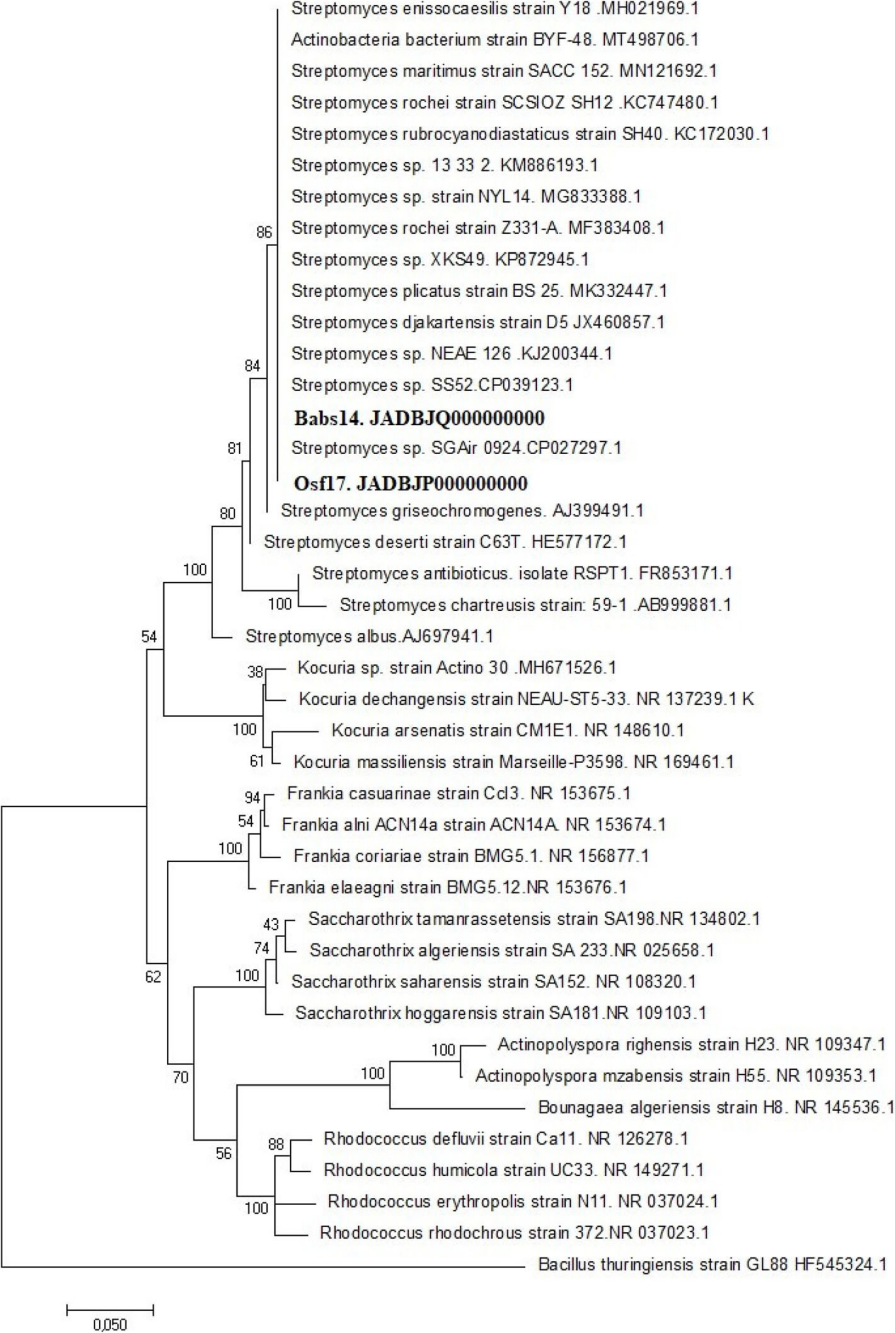
Molecular Phylogenetic analysis of the 16S rRNA sequences of strains Babs14 and Osf17. The tree was constructed by MEGA7 using the Maximum Likelihood method based on the General Time Reversible model. Gamma distribution was used to model evolutionary rate differences among sites (+G, parameter = 0.4926). The rate variation model allowed for some sites to be evolutionarily invariable [+I] 41.92% of the sites. Bootstrap values are expressed as percentages of 1000 replications and the scale bar indicates 0.05 substitutions per site

Babs14 formed a raised, dry, and beige-cream colony with irregular and white margins and an irregular surface, while Osf17 formed a dry, white-gray, convex, and circular colony with a brown pigment diffusion into the surrounding medium. Both strains showed a clear hyphae structure and their morphology and hypha represent those of typical actinomycetes. The isolates were gram-positive, catalase and phosphate solubilization positive. The growth and survival of bacteria are greatly influenced by temperature and both isolates survived up to 45 °C. However, the optimum growth temperature for Babs14 and Osf17 was ca. 30 °C. The optimum pH for Babs14 and Osf17i is pH 6 and pH 7, respectively. The isolates were also evaluated for their ability to withstand salt stress by growing them at different sodium chloride (NaCl) concentrations. Growth arrest was recorded at 7% NaCl with an optimum growth at 1% (w/v) for both strains (Additional file 1, Fig. S1). Phylogenetic analysis of Babs14 and Osf17 was first conducted by MEGA 7.0 with 16S rRNA sequences to determine their phylogenetic affiliation (Fig. 2). Strains Babs14 and Osf17 were clustered as belonging to the *Streptomyce*s genus. The general features of Babs14 and Osf17 are listed in Table 1.

**Table 1:** General features of the strains *Babs14 and Osf17* according to the minimum information about a genome sequence (MIGS) mandatory recommendation

Item	Description	
	Babs14	Osf17
**Classification**	Domain *Bacteria*	Domain *Bacteria*
	Phylum *Actinobacteria*	Phylum *Actinobacteria*
	Class *Actinobacteria*	Class *Actinobacteria*
	Order *Streptomycetales*	Order *Streptomycetales*
	Family *Streptomycetaceae*	Family *Streptomycetaceae*
	Genus *Streptomyces*	Genus *Streptomyces*
	Species *Streptomyces sp.*	Species *Streptomyces sp.*
**Type strain**	Wild-type strain	Wild-type strain
Gram stain	Positive	Positive
Cell shape	Filamentous	Filamentous
Motility	Non-motile	Non-motile
Pigmentation	Beige-cream	White-grey
Sporulation	Sporulating	Sporulating
Optimum Temperature	30 °C	20–40 °C
Optimum salinity	1%	1%
Optimum pH	6.0	7.0
Catalase	Positive	Positive
Submitted to NCBI	JADBJQ000000000	JADBJP000000000
BioProject ID	PJNA665615	PRJNA665615
Collection date	April 2014	October 2017
Latitude and longitude	30°07′57.9″N 2°10′38.6″W	33°29′32.0″N 6°53′46.7″E
Geographic location name	Algeria: Beni Abbes	Algeria: El Oued Souf
Environment biome	Desert	Desert
Environment material	Soil of sand dunes	Soil of sand dunes
Depth	5–20 cm	5–20 cm
Biotic relationship	Free-living	Free-living

### Screening for potential activities

**Fig. 3:**
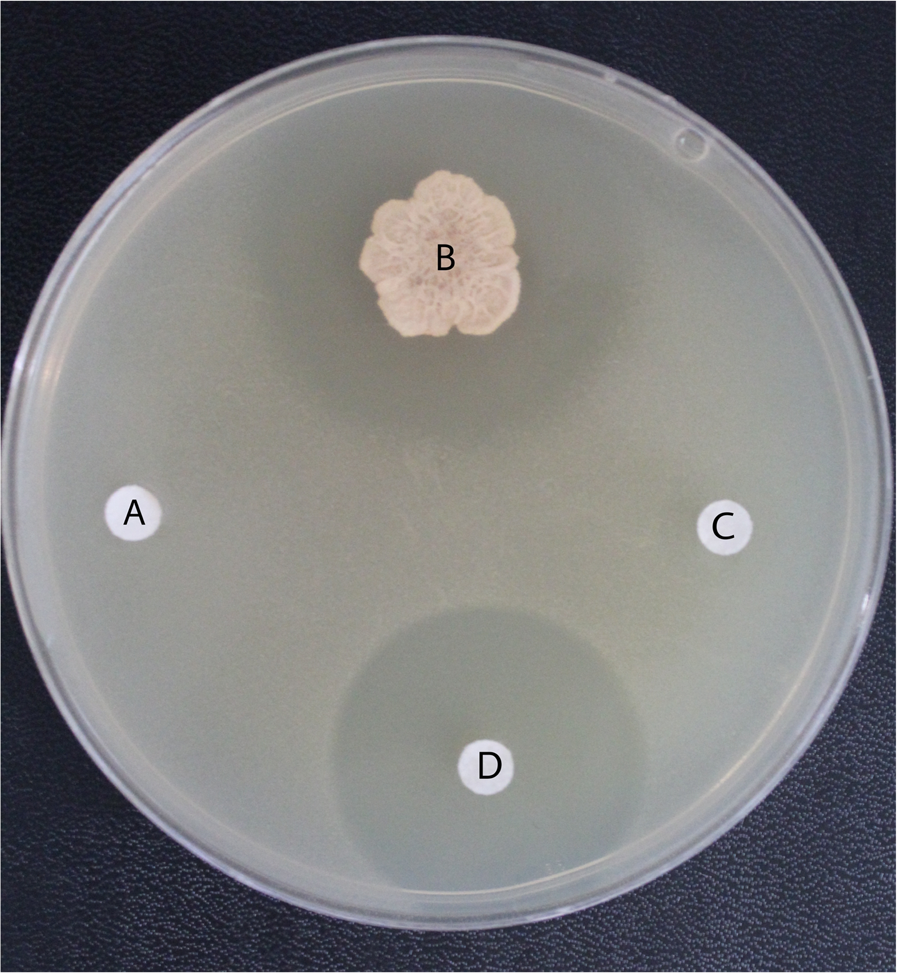
Antimicrobial activity of *Streptomyces* sp. Babs14 against *Bacillus subtilis* subsp. spizizenii DSM 347. **A** negative control (sterile H_2_O), **B** Babs14, **C** ampicillin, and **D** streptomycin

**Fig. 4:**
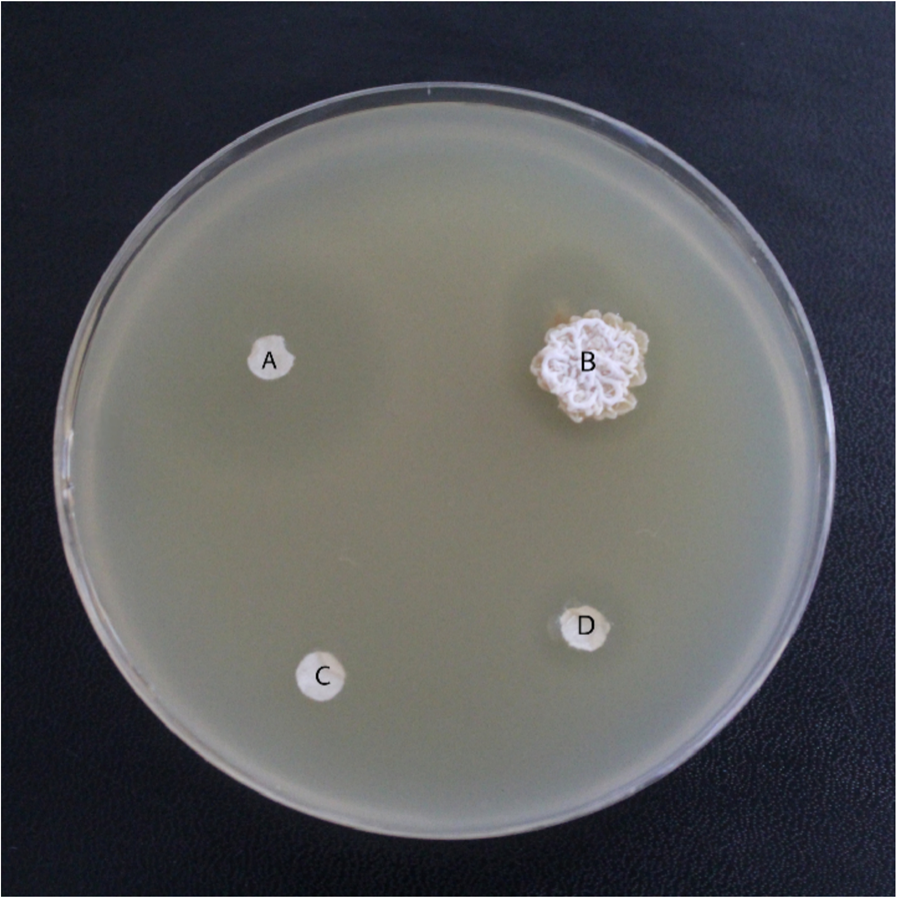
Antimicrobial activity of *Streptomyces* sp. Osf17 against *Escherichia coli* DSM 4899. **A** ampicillin, **B** Osf17, **C** negative control (sterile H_2_O), and **D** streptomycin

Babs14 and Osf17 showed inhibition against all used microorganisms. It was possible to quantify the regions of growth and measure the clear (inhibited) areas, which are indicators of the potency of the antimicrobial compounds. The antimicrobial activities are expressed as the inhibition zone diameter (mm). Strains Babs14 and Osf17 exhibited a clear inhibition activity against all the tested strains (Table 2). The highest antibacterial activity recorded was against *Bacillus subtilis* subsp. spizizenii DSM 347 (Figs. 3, 4) with an inhibition diameter almost similar to the antibiotic streptomycin, which was used as a positive control.

**Table 2:** The inhibition diameter (mm) of the screening of antibacterial, antifungal activities of Babs14 and Osf17

Strains	*E. coli* ATCC 25922	*E. coli* DSM 4899	*S. aureus* ATCC 43300	*B. subtilis* subsp. spizizenii DSM 347	*A. niger* ATCC 6275	*M. ruber* DSM 62748	*M. ruber* van Tieghem DSM 1561
**Babs14**	12 mm	10 mm	15 mm	30 mm	13 mm	18 mm	22 mm
**Osf17**	26 mm	8 mm	20 mm	31 mm	13 mm	16 mm	18 mm

**Fig. 5:**
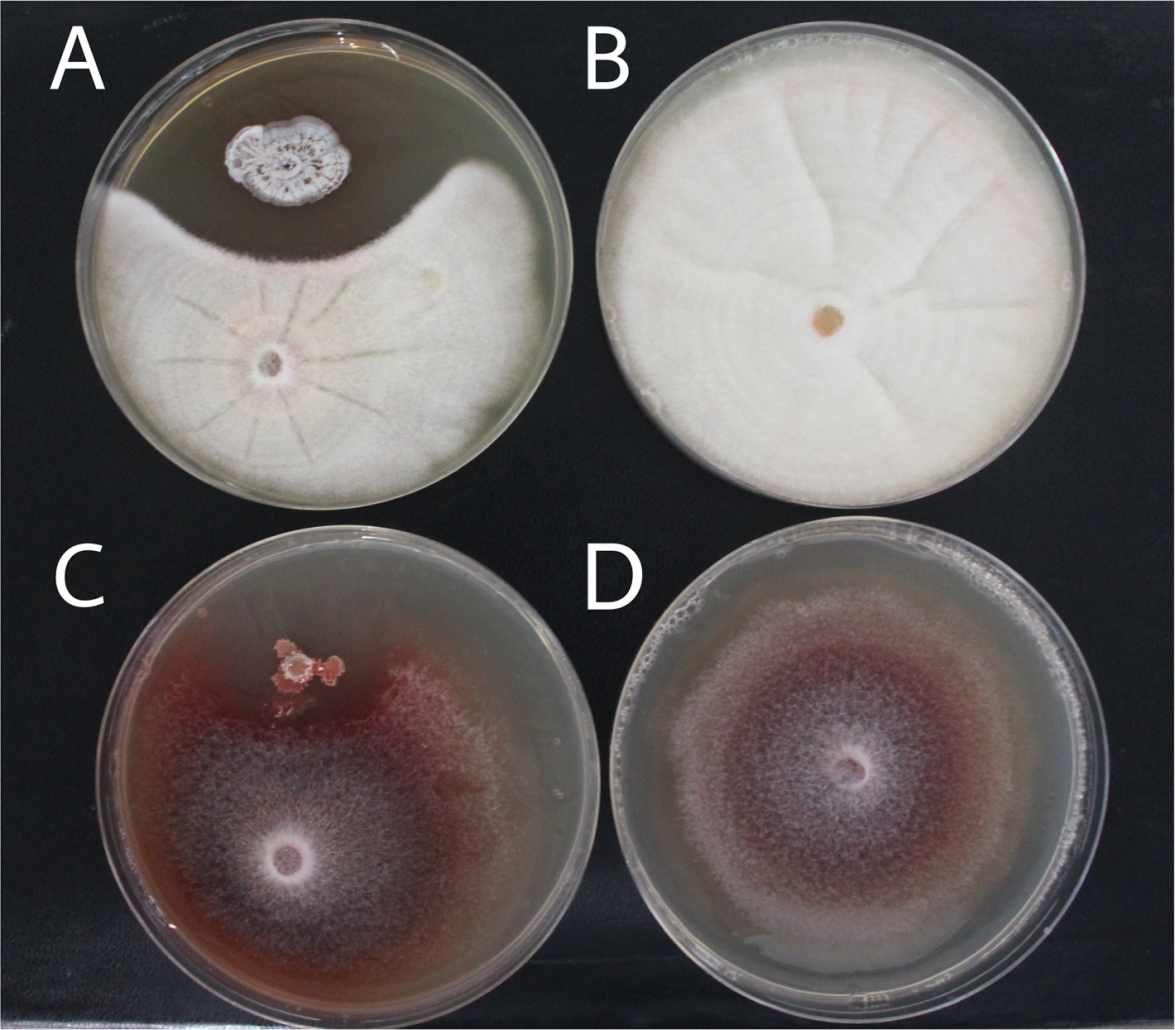
Antifungal activity of *Streptomyces* sp. Osf17 and *Streptomyces* sp. Babs14 by direct streak method against *Monascus ruber* DSM 62748 and *Monascus ruber* van Tieghem DSM 1561 on Malt-agar. Dual bioassay of *Streptomyces* sp. Osf17 against (**A**) *M. ruber* DSM DSM 1561, and (**B**) *M. ruber* DSM 1561. Dual bioassay of *Streptomyces* sp. Babs14 against (**C**) *M. ruber* DSM 62748, and (**D**) *M. ruber* DSM62748

Antifungal activity was tested against *Aspergillus niger* ATCC 6275, *Monascus ruber* DSM 62748, and *Monascus ruber* van Tieghem DSM 1561. Both Babs14 and Osf17 showed inhibition activity against all the tested fungi. The plates were compared with the negative controls, which were performed by growing the fungi without the Saharan strains (Fig. 5).

Enzymatic screening showed a wide enzymatic activity pattern in Babs14 and Osf17. The strains were able to degrade casein, lipid, starch, pectin, cellulose and birchwood xylan. The only difference between the two strains was the absence of detectable proteolytic activity in Osf17 (Table 3).

**Table 3:** Diameter of the clear zone (mm) of the enzymatic assays

Strains	Proteolytic	Lipolytic	Amylolytic	Pectinolytic	Cellulolytic	Xylanolytic
**Babs14**	24 mm	10 mm	12 mm	14 mm	16 mm	10 mm
**Osf17**	–	8 mm	14 mm	20 mm	12 mm	12 mm

### General genome features of Babs14 and Osf17

The whole genomes of Babs14 and Osf17 were sequenced by the PacBio platform and gene completeness was checked by Busco [22]. Our results showed that 92.6% of single copy Busco’s genes were found for Babs14 (C:92.6%[S:92.6%,D:0.0%],F:2.7%,M:4.7%,n:148), and 94.6% were found for Osf17 (C:94.6%[S:94.6%,D:0.0%],F:1.4%,M:4.0%,n:148). Moreover, 0% of Busco’s genes were duplicated in the two genomes. Assembly of Babs14 by Flye 2.4.1 was generated from 2,185,080,676 total bases with a read depth of 266 over the genome and read length N50/N90 was 11,022/6592. For Osf17, the assembly was generated from 2,269,979,737 total bases with a read depth of 275 over the genome and read length N50/N90 was 11,555/6692. The total genome size of Babs14 was 7,999,102 bp with a guanine-cytosine content (GC) of 72.6%. Babs14 assembly resulted in two contigs of 114,649 bp and 7,884,453 bp with a coverage of 298 and 266, respectively.

Strain Osf17 had a genome size of 7,967,258 bp with a GC content of 72.6%. The assembly resulted in two contigs of 7,883,883 bp and 83,375 bp with a coverage of 275 and 306, respectively.

The two genomes are linear, with a topology structure of the contig graph *,1,2 for Babs14 and 4,*,6 for Osf17. The assembly graphs of Babs14 and Osf17 are shown in Additional file 1, Figures S9 and S10.

### Genome annotation

The genome annotation of Babs14 with Prokka version 1.11 identified 6910 protein-coding genes (CDSs), a single tmRNA, 87 tRNA and 18 rRNA genes, and two CRISPR regions. No plasmids were found. Strain Osf17 had 6894 protein-coding genes, a single tmRNA, 86 tRNA and 18 rRNA genes, two CRISPR regions and three native plasmids (Table 4). The BlastN run of the complete genome of Babs14 and Osf17 was used to find the closest relatives based on the highest similarity percentage to the Saharan bacteria. The closest strains to Babs14 and Osf17 were *Streptomyces* sp. SGAir0924 (CP027297.1) and *Streptomyces* sp. SS52 (CP039123.1), respectively, with 99% identity. Average nucleotide identity (ANI) searches were performed using the Kostas lab ANI calculator (http://enve-omics.ce.gatech.edu/ani/index) [23], and the results were confirmed with the JSpeciesWS server (http://jspecies.ribohost.com/jspeciesws/) [24] against the reference genome sequences. The ANI results, together with the BlastN results, were used to reinforce the taxonomic identification, which resulted in an ANI match with over 95% cutoff. Strain Babs14 showed an ANI value of 98.66% with *Streptomyces* sp. SGAir0924 (CP027297.1), which was isolated from outdoor air in Singapore [25], and an ANI of 98.77% was observed between Osf17 and *Streptomyces* sp. SS52. (CP039123), an endophytic strain isolated in Vietnam [26].

**Table 4:** Genome features of *Streptomyces* sp. strains Babs14 and Osf17

Feature	Chromosome characteristics	
	Babs14	Osf17
Genome topology	Linear	Linear
Chromosome size (bp)	7,999,102	7,967,258
GC content (%)	72.6	72.6
Protein-coding genes	6910	6894
Contigs	2	2
rRNA genes	18	18
tRNA genes	87	86
tmRNA genes	1	1
Plasmids	0	3
Pseudo genes (total)	163	189
Secondary metabolite gene clusters BGCs	29	29
Genes assigned to COG	1865	1852
Genes assigned to KEGG	3121	3099
Features assigned by RAST	7261	7241
Gene entries assigned to CAZy	372	370

**Fig. 6:**
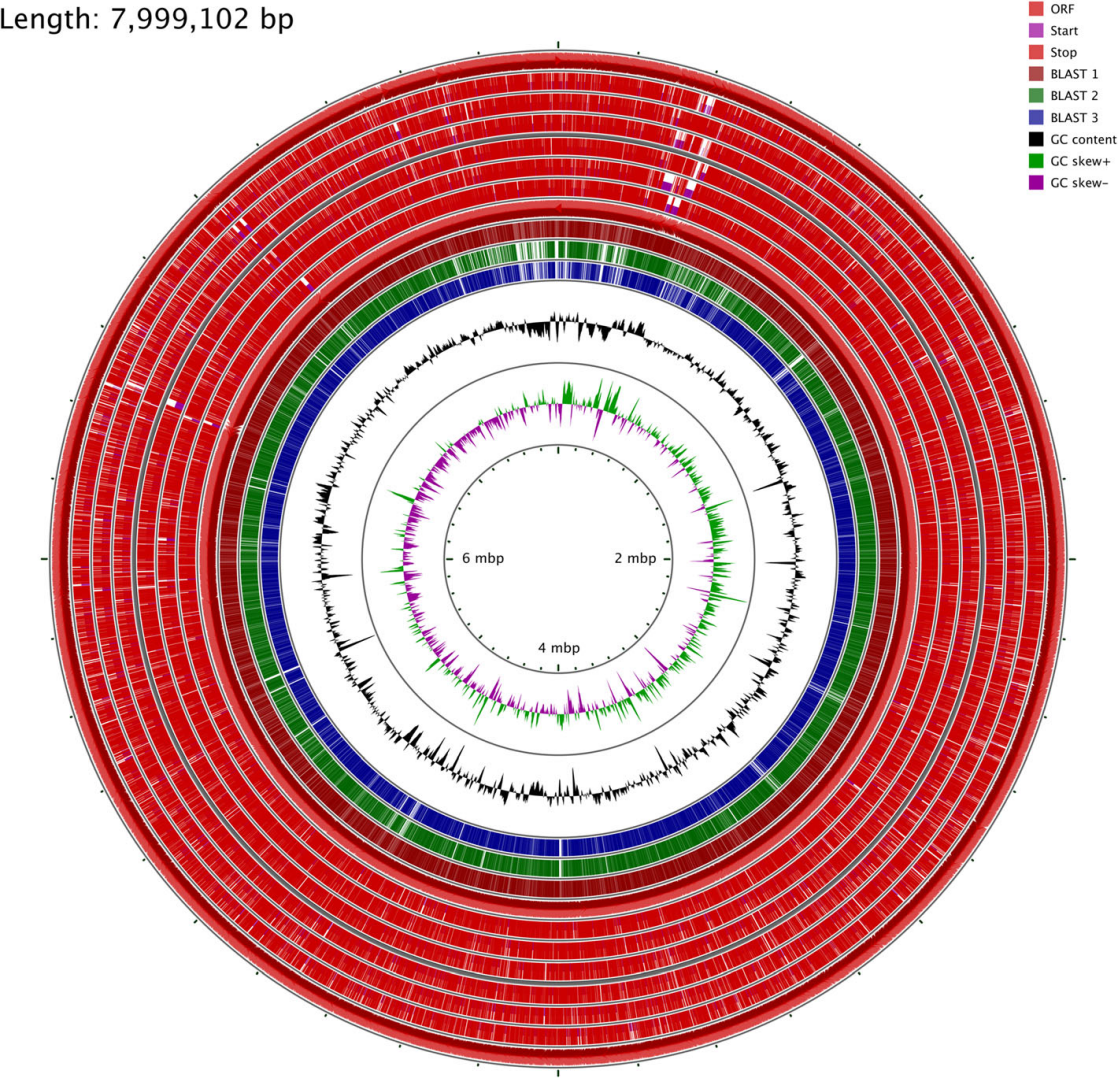
Genomic map of the Babs14 genome as a reference sequence blasted with Osf17, *Streptomyces sp.* SGAir 0924 and *Streptomyces sp*. SS52 using CGViewer Server V1.0. From outside to inside: Ring 1 is the open reading frame (ORF) of the forward strand. Rings 2, 3 and 4 indicate the start and stop codons of the forward strand of Babs14. Rings 5, 6 and 7 indicate the start and stop codons of the reverse strand of Babs14. Ring 8 is the ORF of the reverse strand of the primary sequence of Babs14. Ring 9 corresponds to the Blast 1 of the hits of Babs14 with Osf17. Ring 10 indicates Blast 2 and shows the hits with *Streptomyces sp*. SGAir 0924 (CP027297.1). Ring 11 is Blast 3 and corresponds to the hits with *Streptomyces sp*. SS52 (NZ_CP039123). Ring 12 and 13 show GC content and GC skew, respectively, of the reference sequence Babs14. The map shows the Blast comparison result (BLASTN) with the primary DNA sequence of Babs14. The BLAST results are drawn at partial opacity. The darker regions indicate multiple hits to the corresponding region of the reference sequence Babs14

The genomic map comparison (Fig. 6) using the CGViewer webserver [27] showed that the differences between Babs14 and the three other strains were mostly evenly distributed and there only exists a few areas with larger differences. SEED Viewer maps show that Babs14 and Osf17 are much more similar to each other than with *Streptomyces* sp. SGAir 0924 (CP027297.1) and *Streptomyces* sp. SS52 strains (CP039123) (Additional File 1, Figs. S2-S7). The RAST maps showed that the protein sequence identity between Babs14 and Osf17 was 99% over the entire genome. Moreover, the RAST maps also showed that there are only a few regions below 80% protein identity between the Sahara strains and their closest relatives (Figs. S2-S4). This indicates that the two Saharan strains may have ecological reasons for their genetic similarity despite the extended distance between their isolation site and phenotypic differences. The dot plot comparison based on the genomic sequence of Babs14 and Osf17 showed that the genome sequences are divided in a different way at the genome ends (Fig. S5). Comparison of the DNA sequences of Babs14 and Osf17 to their closest relatives is presented in Additional file 1 (Fig. S6 and Fig. S7). Previous studies on *Streptomyces* genomes have shown that the central region of the genome is conserved and includes mostly housekeeping genes, whereas the dispensable genes are located in the chromosomal arms [28, 29]. The horizontal acquisition of DNA by insertion or deletion events takes place at the ends of the genome [30].

### Analysis of the predicted proteins

**Fig. 7:**
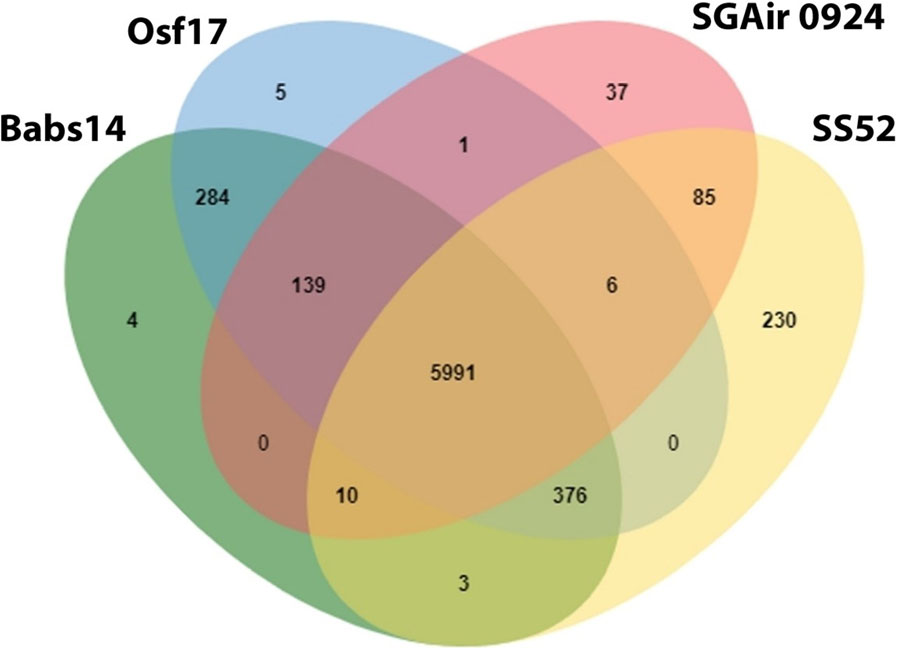
Venn diagram of the number of shared and unique proteins between Babs14, Osf17 and the closest strains *Streptomyces* sp. SGAir0924 (CP027297.1) and *Streptomyces* sp. SS52(NZ_CP039123), as created by OrthoVenn2

OrthoVenn2 (http://www.bioinfogenome.net/OrthoVenn/) [31] was used to compare the proteins of Babs14 and Osf17 and the proteins of their closest relatives, using the files generated by Prokka analysis (Fig. 7). In total, 6807 and 6802 orthologous protein clusters were found in Babs14 and Osf17, respectively. Altogether, 293 protein clusters (284 common to Babs14 and Osf17, and 4 or 5 that are specific to each strain in the Venn diagram) were unique to Babs14 and Osf17. In addition, 39 singleton proteins were found in Babs14 and 48 singleton proteins were found in Osf17 (Table 5). The 284 proteins shared by Babs14 and Osf17, but not found in the other strains, are responsible for different biological functions, such as DNA modification, RNA metabolic process, response to stimulus, carbohydrate metabolic process and many other functions (Additional file 2, Table S1). The OrthoVenn2 analysis indicated that although most of the proteins are very similar, there are clear genetic differences between all the studied strains. Cluster 6455 was unique to Babs14 and Osf17 and is identified as a heat response protein (GO:0009408). In Gene Ontology (GO) term, the heat response protein is assigned to any changes in the activity of a cell or an organism, including enzyme production, secretion, gene expression etc., caused by a heat stimulus [32]. This property is apparently important in the desert environment.

**Table 5:** Orthologous protein clusters and singletons of the Sahara strains and the closest relatives from OrthoVenn2 analysis

Strain	Proteins	Clusters	Singletons
Babs14	6910	6807	39
Osf17	6894	6802	48
*Streptomyces sp*. SGAir_0924	6603	6269	288
*Streptomyces sp*. SS52	7152	6701	363

Both strains showed biological ability for the degradation of different substrates, such as starch, gelatin, xylan, cellulose and pectin. However, an absence of protease (caseinase) activity was observed during screening in Osf17, and a genomic investigation was carried out to better understand this absence. Comparison of the protein sequences showed that both strains have 23 proteases in common: 25 proteases were found in Babs14 and 24 proteases in Osf17, and the carboxy-terminal processing protease CtpA precursor was missing in Osf17. Moreover, three modulators of FtsH and protease HflK were found in Babs14, and only two sequences of the modulators FtsH, and protease HflK were found in Osf17. Furthermore, one protease HtpX was unique to Osf17. The differences between the proteases and the missing sequences, and possibly differing regulation, could be related to the potential inability of Osf17 to degrade casein. It is also possible that the testing time or conditions were not suitable for inducing protease enzyme activity in Osf17.

Enzymatic activities were searched at the sequence level using the Prokka and CAZy annotations and the NCBI Prokaryotic Genome Annotation Pipeline (PGAP). The search confirmed the presence of all the screened activities during the biological tests. Two alpha-amylases were assigned to both Osf17 (locus tags IMX11_33595 and IMX11_10565) and Babs14 (IMX12_34100, IMX12_11065).

Four cellulase family glycosyl hydrolases (locus tags IMX12_32655, IMX12_02660, IMX12_30815 and IMX12_02740) were found in Babs14, and locus tags IMX11_32150, IMX11_02175, IMX11_02255 and IMX11_30305 were found in Osf17. Three lipase sequences were assigned to locus tags IMX11_28100, IMX11_31850 and IMX11_04170 in Osf17, and to locus tags IMX12_28595, IMX12_32355 and IMX12_04665 in Babs14. Only one pectin esterase was found in Babs14 (IMX12_09325) and Osf17 (locus tag IMX11_08825). Two xylanases were found in Babs14 (locus tags IMX12_32005 and IMX12_26840) and in Osf17 (locus tags IMX11_26345 and IMX11_31495). This brief comparison shows that the enzymes of the two Saharan strains are highly similar at the protein level.

### Cluster of orthologous groups (COG) annotation

**Fig. 8:**
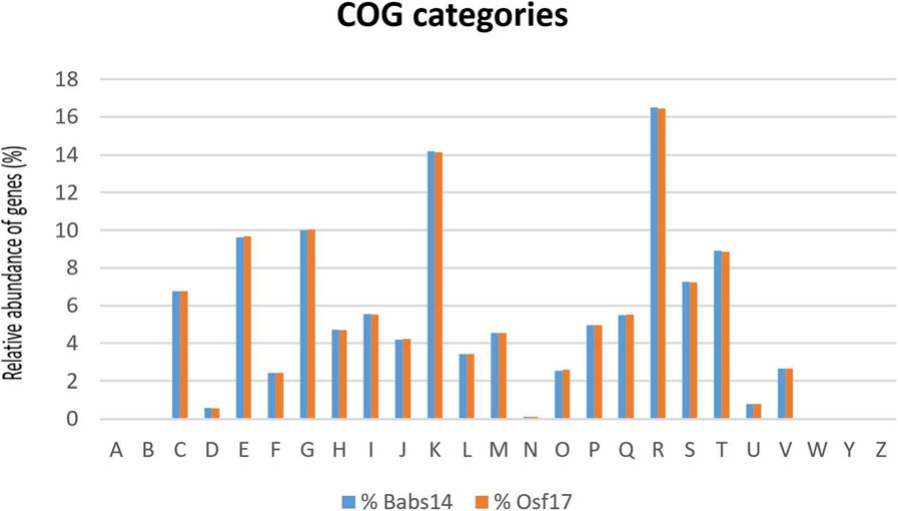
Cluster of Orthologous Groups (COG) database annotation of Babs14 and Osf17. The relative abundance of proteins (%) in the two genomes is shown. Letter codes are described in Table 6

A total of 1865 and 1852 genes were assigned to the COG databases for Babs14 and Osf17, respectively. The numbers of genes annotated by COG were similar in the two strains (Fig. 8; Table 6; see also RAST result in Fig. S8); the genes that encode transcription accounted for the largest proportion of total genes in both Babs14 (14.20%) and Osf17 (14.13%). The genes that encode carbohydrate transport accounted for 10.01% in Babs14 and 10.04% in Osf17. Amino acid transport and metabolism accounted for 9.63% in Babs14 and 9.68% in Osf17 (Fig. 8). The RAST annotation showed many subcategory distributions, and most features confirmed the COG analysis. The largest features were attributed to the amino acids and derivatives, followed by carbohydrate metabolism proteins, protein metabolism and cofactors, vitamins, prosthetic groups, and pigments for Babs14 and Osf17 (Fig. S8). These annotations indicated the ability of these two strains to use the carbohydrates, amino acids, and protein resources available in their living environment.

### Island pathways and phages

Genomic islands (GEIs) could be related to a variety of functions, and play a major role in genome plasticity, evolution, and biological adaptability. Part of the horizontal gene transfer is facilitated by GEIs, who are also implicated in antibiotic resistance, pathogenicity, symbiosis, xenobiotic degradation, and primary and secondary metabolism [33, 34].

Search of the GEIs was performed by the alignment of Babs14 and Osf17 against the reference genomes, *Streptomyces* sp. SGAir0924 (CP027297.1) and *Streptomyces* sp. SS52 (CP039123). In total, 12 GEIs were predicted in Babs14, with an average length of 17,051 bp, and 13 GEIs were predicted in Osf17, with an average length up to 17,741 bp (Table S2). Some CDS sequences were predicted as hypothetical proteins. However, the majority of defined CDSs in the GEIs of Babs14 and Osf17 were related to DNA transcription or response to regulators, e.g., metal-sensitive transcriptional and the TetR family of regulators (TFRs). The latter is widely associated with antibiotic resistance, the control of genes involved in metabolism, and quorum sensing [35]. Sigma factors, phosphate ABC transporters, and enzymes, e.g., hydrolase, oxidoreductase, and metallopeptidase, were also found in the GEIs of Babs14 and Osf17. Moreover, transposases, excisionases and site-specific integrases were predicted in the GEIs. The transposases are associated with genome plasticity, thereby enabling the strain to horizontally transfer genes [36].

Phages were investigated using PHASTER, and one incomplete phage region with a score of 40% was assigned to the *Rhodococcus* REQ1 phage (NC_016655) in the second contig of Babs14. In the first contig of Osf17, three incomplete phage regions with a score of 30% were assigned to the *Bacillus* G phage (NC_023719), *Gordonia nymphadora* phage (NC_031061) and *Erwinia* phage vB_EamM EarlPhillipIV (NC_031007) (Table S3).

### Genetic basis for secondary metabolites

AntiSMASH predicted the presence of different types of biosynthesis gene clusters (BGCs) that encode for potential secondary metabolites. The BGCs of Babs14 and Osf17 are listed in Table S4 and Table S5 (Additional file 2), respectively. In total, 29 biosynthetic gene clusters were predicted in each strain, among which 26 clusters showed similarity to BGCs with a known function. Eight gene clusters were identified containing 100% of the genes from the known cluster (Table 7). The gene clusters of ectoine, SapB, geosmin and hopene are frequently found in *Streptomyces* strains [37, 38]. Ectoine provides protection against osmotic stress and serves as a versatile nutrient [39], and SapB is a morphogenetic peptide involved in growth in a complex medium and is considered as an antibiotic-like molecule [40]. Some clusters were assigned to BGCs based on a possible structural and functional homology with the known antimicrobial molecules, e.g., cluster 2 for candicidin, cluster 13 for albaflavenone and cluster 28 for streptothricin [41–43]. The antibacterial activity displayed by Babs14 and Os17 during the biological tests could be related to the synthesis of these metabolites.

**Table 6:** Cluster of Orthologous Groups (COG) functional classes. Abbreviations for Fig. 8

Functional class	Class description
A	RNA processing and modification
B	Chromatin structure and dynamics
C	Energy production and conversion
D	Cell cycle control /cell division / chromosome partitioning
E	Amino acid transport and metabolism
F	Nucleotide transport and metabolism
G	Carbohydrate transport and metabolism
H	Coenzyme transport and metabolism
I	Lipid transport and metabolism
J	Translation / ribosomal structure and biogenesis
K	Transcription
L	Replication / recombination, and repair
M	Cell wall/membrane/envelope biogenesis
N	Cell motility
O	Posttranslational modification / protein turnover/ chaperones
P	Inorganic ion transport and metabolism
Q	Secondary metabolites biosynthesis, transport, and catabolism
R	General function prediction only
S	Function unknown
T	Signal transduction mechanisms
U	Intracellular trafficking / secretion, and vesicular transport
V	Defense mechanisms
W	Extracellular structures
Y	Nuclear structure
Z	Cytoskeleton

**Table 7:** Potential Biosynthetic Gene clusters assigned in Babs14 and Osf17 showing 100% similarity with the known clusters predicted by antiSMASH

Region	Type	Most similar known cluster
Cluster 1	terpene	isorenieratene
Cluster 7	ectoine	ectoine
Cluster 13	terpene	albaflavenone
Cluster 17	terpene	geosmin
Cluster 20	terpene	hopene
Cluster 24	NRPS	coelichelin
Cluster 25	NRPS	coelibactin
Cluster 27	lanthipeptide	SapB

Furthermore, the hits of clusters 8, 9, 11, 14, 19 and 29 in Babs14 and Osf17 with the known gene clusters were below 100% but over 60% (Tables S4–S5). This might indicate an ability for biosynthesis of melanin, deferrrioxamin B / deferrioxamin E, catenulipeptin, spore pigment, calcium dependent antibiotics and fluostatins M-Q.

As an example of below 60% gene similarity to the known BGCs, cluster 21 in Babs14 showed hits with different metabolites (Fig. S11): 44% similarity to sceliphorolactam and 60% to vicenistatin. However, as many essential genes in the metabolic pathways were missing, it is unclear as to the eventual role of the pathway. The BGCs with a low percentage of similarity to known clusters may not represent functional gene clusters, or they may be complemented by genes in other regions. Many gene clusters showed no similarities with known BGCs, some might even be part of other gene clusters as they are composed of one or two genes [44]. Further studies are needed to elucidate the role of these pathways.

From the 29 BGCs predicted in the Saharan strains, 15 are also present in *Streptomyces* sp. SGAir0924 (CP027297.1) and 18 in *Streptomyces* sp. SS52 (CP039123). However, differences between BGCs exist, and previous studies have shown that they could be driven by the evolution and the adaptation of these strains to survive and proliferate under harsh environmental conditions [44].

### Adaptation to environment and stress responses

In extreme and rapidly changing conditions, microorganisms must sense the changes and respond with appropriate mechanisms. Stress response in Babs14 and Osf17 was investigated using SEED viewer version 2.0 [45]. In total, 28 stress response entries were found for both strains, which included osmotic, oxidative, and periplasmic stress responses, and detoxification and dimethylarginine metabolism (Additional file 2, Table S6). Extracytoplasmic function (ECF) factors sigma B (σ^B^) and sigma E (σ^E^) were found in Babs14 and Osf17. These factors are involved in gene regulation and expression in response to various extracellular changes. Sigma E is involved in the control of the periplasmic heat shock regulation, which is activated by unfolded proteins in the periplasm [46]. Sigma B plays a role in resistance to a variety of stressors, such as high and low pH, heat, high osmolarity, high ethanol concentrations and oxidizing agents [47, 48]. The peroxide stress protein YaaA is involved in the cellular response to hydrogen peroxide stress, and it prevents oxidative damage to the DNA and proteins. This protein was predicted in both Babs14 (IMX12_11470) and Osf17 (IMX11_10970).

Two protein sequences were predicted from the gene *groL:* locus tags IMX12_16460 and IMX12_22000 for Babs14, and locus tags IMX11_15960 and IMX11_21500 for Osf17. These proteins are related to the 60 kDa chaperone family and promote refolding of misfolded polypeptides, especially under stressful conditions [49].

In addition to the universal stress proteins, calcium homeostasis/redox stress proteins were predicted in Babs14 (locus tag IMX12_11785) and Osf17 (locus tag IMX11_11285). The GlsB/YeaQ/YmgE family stress response related to the membrane was found in Babs14 (IMX12_04415, IMX12_08985, IMX12_18995, IMX12_21360) and in Osf17 (locus tag IMX11_03925, IMX11_08485, IMX11_18495, IMX11_20860). The alkaline shock Asp23/Gls24 family that envelope stress response was also predicted in Babs14 (locus tagIMX12_09080) and Osf17 (IMX11_08580), as well as 50S ribosomal protein L25/general stress protein Ctc in Babs14 (IMX12_15415) and Osf17 (IMX11_14915).

Genome annotation confirmed the presence of heat shock proteins (Hsp) in each Sahara strain including: Hsp 15, Hsp18, heat-inducible transcription repressor HrcA and putative heat shock protein HspR. Previous studies on *Streptomyces albus* have shown that HSP18 is involved in thermotolerance at extreme temperatures [50]. Chaperone proteins ClpB, DnaK, DnaJ and HtpG were found in Babs4 and Osf17, which are part of the stress-induced multi-chaperone system involved in the recovery of cells from heat-induced damages [51].

During winter, the temperature in the Algerian Sahara can drop to below freezing. As such, cold shock responses were investigated. Cold-shock proteins (Csp) are induced upon temperature downshift and are involved in the adaptation of cells to cold. Six proteins were predicted as **Csp** in Babs14 (locus tags IMX12_16345, IMX12_16465, IMX12_18970, IMX12_20825, IMX12_21625 and IMX12_32830) and in Osf17 (locus tags IMX11_15845, IMX11_15965, IMX11_18470, IMX11_20325, IMX11_21125 and IMX11_32325). One cold shock domain-containing protein was assigned to Babs14 (IMX12_22005) and one to Osf17 (IMX11_21505). Cold shock protein ScoF, cold shock-like protein 7.0 and CspA, which is a major shock protein, were found in both strains. Recent studies have shown that Csps might also have a wider role in stress tolerance of bacteria [52].

Programmed cell death and toxin-antitoxin systems Phd-Doc and YdcE-YdcD were found in Babs14 and Osf17, and many studies have suggested that they can activate programmed cell death to survive in different environmental stresses caused by nutrient deprivation and antibiotics. Toxin-antitoxin systems have also been shown to be responsible for stress management, bacterial persistence, plasmid maintenance, and biofilm formation [53].

### Comparison of Babs14 and Osf17 with other Streptomyces strains isolated from the desert

**Fig. 9:**
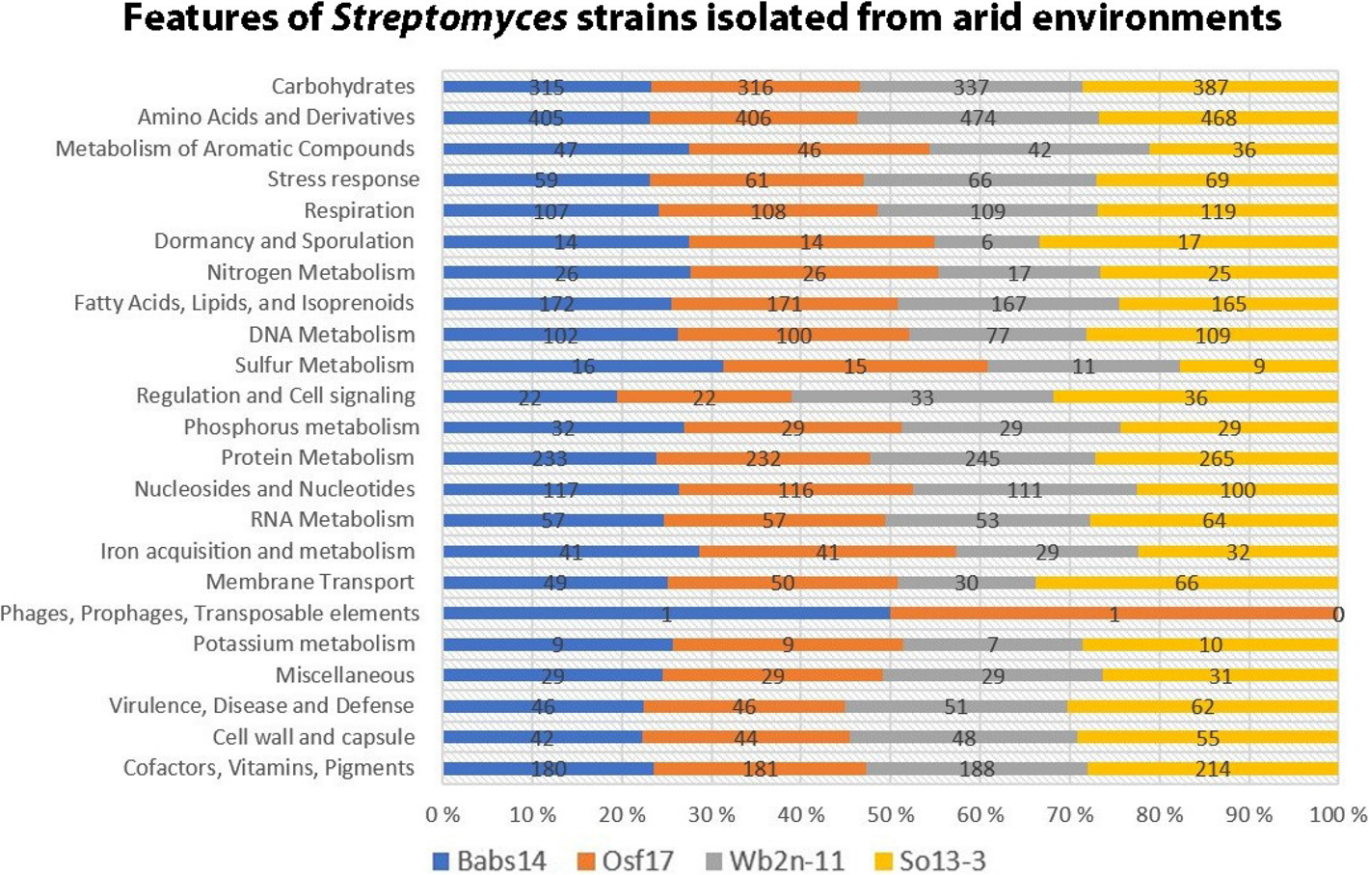
Features of *Streptomyces* sp. Babs14 and Osf17 isolated from Sahara and *Streptomyces* sp. Wb2n-11, and So13–3 isolated from Sinai desert and Antarctic cold desert using the SEED subsystem

Comparison between the general features of the selected strains using RAST showed a similarity in the distribution of many subsystems between strains Babs14, Osf17, *Streptomyces fildesensis* So13.3 (CP048835.1), and *Streptomyces* sp. strain Wb2n-11 (CVPB00000000), even if the genome sizes were not similar (Fig. 9). However, a greater number of genes were implicated in the metabolism of aromatic compounds (the second most widely distributed class of organic compounds in nature after carbohydrates [54]) in Babs14 and Osf17. The ability to degrade aromatic compounds is usually coded by mobile genetic elements, which facilitates their horizontal gene transfer for rapid adaptation of microorganisms [55]. Moreover, the anaerobic degradation of aromatic compounds was only predicted for Babs14 and Osf17 and was assigned to the bacterial non-oxidative, reversible multi-subunit hydroxyarylic acid decarboxylases/phenol carboxylases, which are encoded by the three clustered genes (B, C, and D) with approximately 0.6, 1.4, and 0.2 kb sizes, respectively [56].

The nitrogen metabolism predicted in Babs14 and Osf17 included nitrosative stress. The nitrite-sensitive transcriptional repressor NsrR, which is the major bacterial nitric oxide stress sensor, was only found in Babs14 and Osf17. In *Streptomyces coelicolor*, NsrR is a dimeric Rrf2 family protein that reacts rapidly with up to eight molecules of nitric oxide [57]. Furthermore, four denitrification gene clusters were present only in Babs14 and Osf17: respiratory nitrate reductase alpha chain (EC 1.7.99.4), nitrate reductase beta chain (EC 1.7.99.4), respiratory nitrate reductase delta chain (EC 1.7.99.4), respiratory nitrate reductase gamma chain (EC 1.7.99.4), which were missing in the other strains. Fatty acid, lipid and isoprenoid metabolism predictions showed a greater number of subsystems due to the larger proportion of isoprenoids; 51 in Babs14 and Osf17, 12 in S*treptomyces* sp. So13.3 (CP048835.1), and 37 in *Streptomyces* sp. Web2n-11 (CVPB00000000).

Iron acquisition and metabolism was also more widely represented in the genome, especially due to the number of proteins associated with siderophores. Concentrations of major elements (such as aluminum and iron) and minor elements (phosphorus) are high in Saharan soils and dusts [58]. Most of the phosphorus in Saharan soil is present in inorganic form and is associated with iron oxyhydroxides coated on clay minerals and quartz particles [59]. The presence of proteins associated with nitrogen, phosphorus, and iron metabolism in Babs14 and Osf17 could be related to the abundance of these elements in their environment (Fig. 9). The presence of phage and transposable elements was only observed in the Saharan bacteria Babs14 and Osf17.

The antiSMASH analysis predicted 29 BGCs for Babs14 and Osf17, 30 BGCs for *Streptomyce*s sp. So13–3 (CP048835.1), and 34 for *Streptomyces* sp. Wb2n-11(CVPB00000000). Melanin, ectoine and Sap B were predicted for all strains. However, geosmin and spore pigment were missing in *Streptomyces* sp. Web2n-11 (CVPB00000000). The presence of indole was only predicted in Babs14 and Osf17. The highest number of terpenes was predicted in the Saharan strains; seven clusters for Babs14 and Of17, and three for the other strains.

The OrthoVenn web platform was used for comparison and annotation of orthologous gene clusters among the selected strains. *Streptomyces* sp. Wb2n-11(CVPB00000000) and *Streptomyces* strain So13–3 (CP048835.1) showed the highest number of proteins and singletons. However, Babs14 and Osf17 had the highest number of orthologous protein clusters, 6783 and 6776, respectively, and the lowest number of singletons. Many clusters were shared by the Saharan strains and the other *Streptomyces* strains. In total, 229 proteins were unique to the Saharan strains Babs14 and Osf17. Many protein clusters unique to Babs14 and Osf17 were related to the metabolic process. Moreover, two clusters were assigned to the response to toxic substances GO:0009636 and antibiotics GO:0046677. One of the most interesting clusters was cluster7718, which is associated with DNA mediated transformation GO:0009294. Cluster 7741 and cluster 7784 were related to DNA-mediated transposition GO:0006313.

**Fig. 10:**
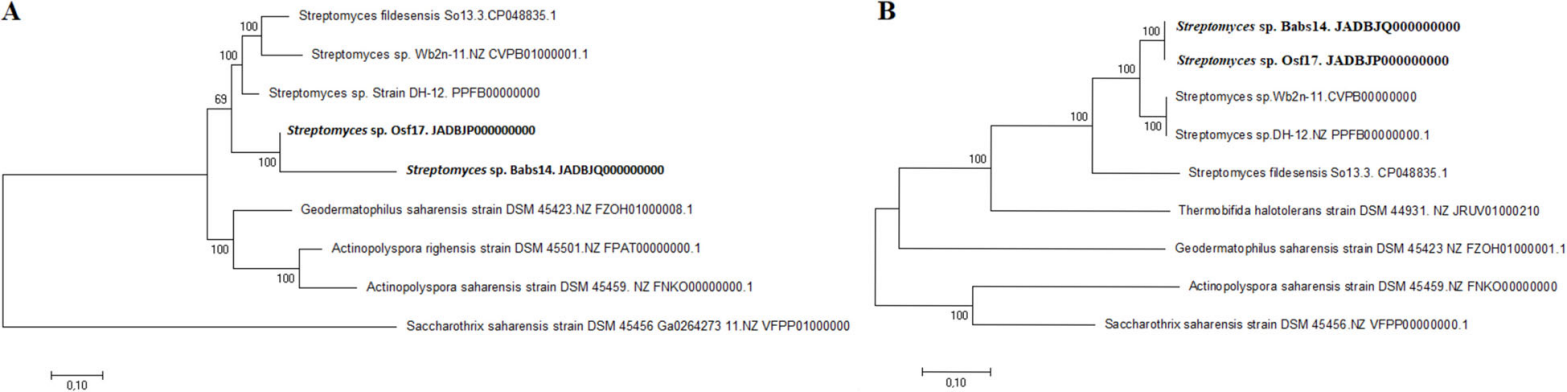
Phylogenetic analysis of actinomycetes isolated from arid areas. **A** Phylogenetic tree of the concatenate housekeeping genes *atpD*, *gyrB*, *recA*, *rpoB* and *trpB* based on the Maximum Likelihood method and the GTR model with Gamma distribution. **B** Phylogenetic analysis of the concatenate proteins *RuvA, RuvB,RuvC, RecG* based on the Maximum Likelihood method using a JTT model with a discrete Gamma distribution (+G, parameter = 0,6118). The scales indicate 0.1 substitution per site

Analysis of housekeeping genes *atpD*, *gyrB*, *recA*, *rpoB* and *trpB* has been used to predict the diversity of the functional potential within a species, and to clarify relationships between closely related strains [60]. It was noted that Babs14 and Osf17 are phylogenetically distant from the other *Streptomyces* strains used in this analysis and appear on a well-supported clade in the phylogenetic trees (Fig. 10 A).

Homologous genetic recombination promotes genetic diversity and plays an important role in repairing different types of DNA damage [61] The key intermediate formed during homologous recombination is the Holliday junction that is formed after the RecA-mediated exchange of DNA strands. The *RuvABC* process holiday junctions and are critical to bacterial DNA repair [62], and *RuvABC* and *RecG* are known to be well-conserved among *Streptomyces* species [63]. The analysis of the protein sequences of these genes, for the strains isolated from arid areas, including *Streptomyces* sp. Babs14 and *Streptomyces* sp. Osf17, resulted in a phylogenetic tree in which these strains appear on the same clade distant from the other desert *Streptomyces* species (Fig. 10 B). Previous research has shown that DNA repair in bacteria might contribute to the evolution of bacterial genomes by participating in the acquisition of foreign DNA from distantly related organisms during horizontal gene transfer events and is most likely related to bacterial adaptation [64].

## Discussion

*Streptomyces* strains are of a great commercial importance due to their biologically active compounds. Previous reports have demonstrated that actinobacteria residing in deserts have the capability to survive under extreme conditions and possess many gene clusters to produce bioactive compounds [65]. Connecting the natural abilities of active metabolite production to the genes that encode them has enabled the utilization of genome sequence data in the discovery of new molecules. The antagonistic potentials of actinomycetes isolated from the Algerian desert against plant pathogenic fungi have been reported [66, 67]. The previous investigation showed the potential of these microbes to produce antifungal compounds but also many hydrolytic enzymes [66, 67]. To investigate further the potential bioactivities in Saharan microorganisms, strains Babs14 and Osf17 were isolated from the sand dunes in Beni Abbes and El Oued Souf, which are arid areas in the Eastern great erg and the Western great erg in Algeria, respectively. The strains were selected for their activity profiles, which include antibacterial, antifungal, and enzymatic activities. Molecular identification allowed us to classify Babs14 as *Streptomyces sp.* with a genomic similarity of 99% to *Streptomyces sp*. SGAir0924, which had been isolated from outdoor air collected in Singapore (a tropical climate with temperatures that average 33 °C). The genomic study of *Streptomyces sp*. SGAir 0924 identified genes involved in stress response regulation, which may be related to desiccation in the air and the survival of this strain under arid conditions [25]. The high similarity of Babs14 to *Streptomyces sp*. SGAir 0924 could be explained by the adaptation of these strains to the challenging climate conditions of their environments. Strain Osf17 showed 99% similarity to *Streptomyces sp.* SS52, a strain isolated from *Phyllanthus urinaria* in Tra Vinh province of Vietnam, and this endophytic strain showed the capacity to produce daidzein in the culture medium [26]. However, the Saharan strains differ from their relatives in many aspects, including secondary metabolites, orthologous genes, and enzymes.

Babs14 and Osf17 were found to genetically resemble each other in terms of enzymes, BGCs and stress response. However, the two Saharan strains differ from each other in a number of aspects, such as plasmids, genomic islands, phages, and some proteins. Based on OrthoVenn2 analysis, 284 proteins were shared by the Sahara strains, which are missing in their closest relatives. These proteins were assigned to different metabolic and cellular functions, in which some proteins could be related to the adaptation of these bacteria to the available resources in their biotope. The metabolic pathways associated with aromatic compounds, nitrogen, phosphorus, and iron acquisition were present in a greater proportion in Babs14 and Osf17 (Fig. 9) and could be related to the abundance of these naturally occurring elements in the Saharan soils [68]. The high genomic similarity of these phenotypically differing strains supports the earlier finding that plant-free deserts show low levels of phylogenetic and taxonomic diversity and low diversity in protein-coding gene categories [11]. The high similarity might reflect their ecological adaptation and their low optimal growth temperature (~ 30 °C) rather than their highest daytime temperatures (~ 45 °C), which probably reflects the environment of these microbes under the soil surface. As such, the sand probably partly protects the microbes isolated in the layers below the surface.

Sand dune habitats are changed by many factors, e.g., dune encroachment, sand accumulation, and wind erosion [69]. Previous studies of dust samples collected from the Saharan region in Cap Blanc showed that dusts contain organic compounds, such as charcoal-like fragments of burnt vegetation, leaf wax-derived lipids adsorbed on clays, pollen grains, and amorphous material [70]. The existence of a wide range of hydrolytic enzyme activities and carbohydrate-active enzymes indicates that these strains are able to survive in sand areas that contain plant debris, e.g., distributed by wind. Dust and sandstorms also play a role in spreading Saharan microbes and spores to various locations [71]. The whole genome sequencing of Saharan microbes offers the possibility to identify the microbes that are spread and thus create a more complete picture of the influence of Saharan microbes in other geographic areas.

Genome mining and metabolite analysis indicate that the isolated strains have a great potential for secondary metabolites production. Previous investigations on BGCs have shown that more than 95% of the genes involved in secondary metabolism reside mostly in the accessory (dispensable and unique) genomes of *Streptomyces* species [72]. This reflects the diversity of secondary metabolism of these strains, and only 5% of genes for secondary metabolism were found in the conserved core genome [72].

Many genes involved in antibiotic biosynthesis show a high similarity with known genes, which suggests that Babs14 and Osf17 and similar strains could be sources for commercially applicable secondary metabolites. It has been reported that the *Streptomyces* genus is abundant in the Atacama Desert and 50% of these strains produce antibacterial and other compounds, such as carotenoids with antibacterial activity, even against gram-negative bacteria [73]. AntiSMASH has been used widely to identify biosynthesis gene clusters, e.g., in *Streptomyces* [74–78]. A genome-wide study showed that *Streptomyces* genomes possess 25–70 known BGCs, but the genomes could contain more biosynthetic gene clusters that code for secondary metabolites [79]. It is also possible that antiSMASH did not identify all potential BGCs. Novel metabolites might be identified by further genome mining with the support of mass spectrometry analysis.

Microorganisms from arid soils are exposed to periodic nutrient starvation and various abiotic and biotic stresses. These conditions are unfavorable for bacterial growth, and to survive, bacteria must respond and adapt. One strategy of desert microbiota is to increase the abundance of genes involved in osmoregulation and dormancy, which contribute to their survival in hostile environments [80]. The stress responses investigated in this study identified the presence of BGCs in Babs14 and Osf17, which could be involved in adaptation to the stress caused by the Saharan environment, e.g., pigments, ectoine, carotenoid, and also factors that respond to heat, cold, and oxidative and osmotic stresses. Spore coats contain pigments that absorb UV radiation and play a significant role in the resistance to UV-A and UV-B radiation [81, 82].

The study of stress response in Babs14 and Osf17 indicates the genetic ability of these strains to resist various stress conditions. However, many genes and their predicted proteins are highly similar (98–99%) to the other *Streptomyces* species. Previous studies on *Rhodococcus jostii* RHA1 (GCA_000014565.1) have shown that desiccation resulted in a transcriptional response eight times greater after air-drying treatment [83]. The genes regulated included *dps1*, which encodes for oxidative stress protection, and the two genes that encode sigma factors SigF1 and SigF3. The transcription, expression, and regulation of these genes in Babs14 and Osf17 under stress conditions requires further study.

The mechanisms implicated in resistance to desiccation and radiation tolerance have no single molecular explanation, and multiple systems could make significant contributions. These mechanisms reflect many interactions at the structural, physiological, and molecular levels. The adaptation of existing DNA repair enzymes, protein oxidation and nucleoid condensation are the main described mechanisms for resistance to desiccation and irradiation [84]. Although, classical DNA repair systems may be shared widely in different resistant and sensitive bacterial species and are quite similar from one bacterial species to another, they are not identical. The differences reflect the lifestyle and environment of each species. The improvements are in the amelioration of protein oxidation, as earlier research has pointed out, although the biochemical processes remain to be explored [84].

The genomic comparison of the Saharan strains Babs14 and Osf17 with seven actinomycetes isolated from hot and cold deserts using the housekeeping gene sequences, resulted in a phylogenetic tree where Babs14 and Osf17 were on the same clade distant from the other desert *Streptomyces* species. Moreover, the phylogenetic analysis of the protein sequence of the genes labelled as *RuvA, RuvB, RuvC* and *RecG* showed differences between the *Streptomyce*s species, and resulted in a distant and separated clade for Babs14 and Osf17. The phylogenetic analysis highlighted the resemblance of Babs14 and Osf17 to each other, but also emphasized the differences with the other desert species.

## Conclusion

Our study of the two Saharan strains revealed the reasons for adaptation to the desert environments. The competitive production of enzymes and secondary metabolites synthesis, including the stress response mechanisms ensure that they can survive in extreme ecological niches [11]. Globally, Sahara is one of the largest deserts and it spreads across several countries of Africa but has one of the smallest registration rates of biodiversity in biological databases [85]. Actinobacteria seem to dominate the microbial desert soil communities [86]. Whole genome sequences from Saharan microbes has received little attention to date [8]. Our study opens the way to investigate more profoundly the microbial diversity, adaptation, and genomic evolution in the desert conditions of Sahara. The genomic data comparison and analysis could increase our understanding of the adaptation capability but also the pathways and enzymes of *Streptomyces* that live in extreme environments, which could have a biotechnical and pharmaceutical significance.

## Methods

### Sampling and microbial isolation

Non-rhizosphere soil samples from 5 to 20 cm depth were collected from Beni Abbas and El Oued Souf. The samples were packed in sterile containers and stored at 4 °C [66]. The samples were air-dried and heated aseptically for 1 h at 45 °C in a hot air oven, then cooled to room temperature to remove the undesired gram-negative bacteria. Selective media were used to promote *Actinomycetes* growth [66]. Dilution techniques and different media, such as yeast extract–malt extract agar medium (ISP2) and starch-casein agar (SCA) were used for the isolation. The plates were incubated at 28 °C to 40 °C for 14 days at pH 7. Colonies were picked out and streaked until purity. All the isolates were examined by light microscopy to detect the actinomycetes and stored at 4 °C for further studies [87, 88].

### Morphological and cultural characterization of the isolates

The cultural characteristics of the isolates were observed on yeast extract malt extract agar (ISP2), oatmeal agar (ISP3), and inorganic salt starch agar (ISP4). The plates were incubated at 30 °C for 7–21 days. The sporulation was observed by light microscopy [66, 89]. The gram stain and phosphate solubilization tests were performed as described in the literature [90, 91]. The catalase activity was determined by adding 3% hydrogen peroxide (H_2_O_2_).

To study the effect of temperature on the growth, the strains were inoculated in 250 mL Erlenmeyer flasks containing 100 mL of liquid media ISP2. The flasks were incubated at different temperatures: 5 °C, 25 °C, 30 °C, 40 °C, 45 °C and 50 °C by shaking at 200 rpm for 7 days. The determination of the growth pH ranges was performed using the liquid medium ISP2 at pH 4, 5, 6, 7, 8, 9,10 and 11, incubated at 30 °C by shaking at 200 rpm for 7 days. Salt concentration has a profound effect on the bacterial growth, especially due its effect on osmotic pressure. To observe this effect, NaCl was added to the liquid medium ISP2 at different concentrations (0, 1, 2, 3, 4, 5, 6, 7%) and incubated at 30 °C by shaking at 200 rpm for 7 days [92]. Each treatment was replicated in triplicate and the optical density (OD) was measured by a spectrophotometer at 600 nm light spectrum. The initial density of each flask after inoculation was subtracted from the final growth measurement [92].

### Screening of the antimicrobial and antifungal activities

Antimicrobial activity was tested in vitro against gram-negative bacteria *Escherichia coli* ATCC 25922, *Escherichia coli* DSM 4899 and gram-positive strains *Bacillus subtilis* subsp. spizizenii DSM 347 and *Staphylococcus aureus* ATCC 43300. Antifungal activity was tested against *Aspergillus niger* ATCC 6275, *Monascus ruber* DSM 62748, and *Monascus ruber* van Tieghem DSM 1561*.*The antibacterial tests were carried out by the agar antimicrobial spot assay. The pure isolates were spot inoculated (6–8 mm) on ISP2 agar and incubated for 5 days at 30 °C. A second layer was made by pouring 10 ml of semi-agar (0.7%) on a plate inoculated with 1.5 10^8^ CFU ml^− 1^ (counted at 600 nm value 0.5 according to the McFarland standard) of the pathogenic microorganisms. The plates were incubated at 37 °C for 24 h. A clear zone around the spot was considered as positive. Two antibiotics were used as positive controls: 10 μl of stock solution of 100 mg/ml of Ampicillin or Streptomycin was added to filter paper (6 mm disc) and then air dried. A filter disc with 10 μl of sterile water was used as negative control [93, 94]. Cross streak and dual culture bioassay methods were used for the screening of the antifungal activity. The isolates were inoculated on the Malt-agar plates and incubated for five days at 30 °C. The pathogenic fungus was inoculated perpendicular to the first spot. The plates were incubated for 48–72 h and compared with the pathogen control plates [95, 96]. The antibacterial and antifungal interactions were analyzed by observing the inhibition zone size, expressed in millimeters [93].

### Enzyme screening

Enzymatic activities, including amylolytic, proteolytic (caseinase), lipolytic, pectinolytic, cellulolytic and xylolytic activities, were screened using zone clearance assays. Gelatin hydrolysis was screened using a nutrient gelatin medium; the tubes were inoculated and incubated at 30 °C for up to 7 days. After incubation, the tubes were kept on ice for 15 to 30 min. Hydrolyzed gelatin results in the liquefaction of the medium, even when exposed to cold temperatures [97]. Enzymatic potential screening was carried out by streaking the strains in spots on agar media containing (g/L): 0.05 g MgSO4·7H2O, 0.05 g NaCl, 0.01 g CaCl2, 0.2 g yeast extract, 0.5 g peptone, 2 g agar [98]. Next, 1% (w/v) of the different substrates was incorporated as a principal carbon source. The plates were incubated for 3–7 days at 30 °C, and then amylase and pectinase plates were flooded with an iodine solution [99, 100]. Xylanase and cellulase plates were stained using Congo red solution and washed after 10 min with 1 M NaCl [101, 102]. A clear zone around the colony indicates the presence of the screened activity. The production of lipolytic enzyme was detected using Sorbitan monolaurate (Tween 20). The indication of lipolytic enzymes in the colony was a visible precipitate due to the formation of crystals of calcium salt of lauric acid liberated by the enzyme [103]. The proteolytic activity was tested qualitatively on casein agar using skim milk; the ability of the bacterial strains to use protein (casein) is shown through clear zones that surround the colonies [104, 105].

### PCR amplification, phylogenetic analysis, and molecular identification

The bacterial agar plates were shipped to Macrogen Europe. Polymerase chain reaction (PCR) was performed by Macrogen Europe to amplify and sequence the 16S rRNA sequences using primers 27F (5′-AGAGTTTGATCCTGGCTCAG-3′) and 1492R (5′-GGTTACCTTGTTACGACTT-3′). The sequences were aligned using the homology search by comparing the sequence with those present in the public database (NCBI) using the standard Basic Local Alignment Search Tool (BLAST). Phylogenetic analysis was conducted using the Molecular Evolutionary Genetics Analysis (MEGA) software version 7 [106] and multiple alignments of data were performed by Clustal W [107]. The phylogenetic tree was reconstructed with the neighbor-joining algorithm. Topology of the resultant tree was evaluated by bootstrap analysis of the neighbor-joining dataset, based on 1000 replications [108].

### Whole genome sequencing

The microbial strains were cultivated in Yeast-Malt-Glucose (YMG) broth for 48 h at 30 °C to produce the cell mass. The genomic DNA of strains Babs14 and Osf17 was initially extracted using Invitrogen PureLink® Genomic DNA kit. The DNA quantity and quality were tested by the NanoDrop ND-1000 Spectrophotometer (Thermo Fisher Scientific). The DNA was purified further using the Quick-DNA Miniprep Plus kit by Zymo research at the sequencing center of the University of Oregon Genomics & Cell Characterization Core Facility (GC3F). The whole genome sequencing was performed there by Pacific Biosciences Sequel II technology (PacBio). The DNA was made into SMRTbell libraries using the Express Template Prep Kit 2.0 from PacBio according to the manufacturer’s protocol. Samples were pooled into a single multiplexed library and size was selected using Sage Sciences’ BluePippin, which uses the 0.75% DF Marker S1 High-Pass 6 kb–10 kb v3 run protocol and S1 marker. A size selection cutoff of 8000 (BPstart value) was used. The size selected SMRTbell library was annealed and bound according to the SMRT Link Set Up and sequenced on a Sequel II. Raw PacBio reads were converted to fasta format with Samtools Fasta (http://www.htslib.org/doc/samtools.html) and then assembled with Flye 2.6 (https://github.com/fenderglass/Flye) with parameters-plasmids-iterations 2-asm-coverage 120.

### Genome annotation and analysis

The assembled bacterial genomes were annotated with Prokka 1.11 (https://github.com/tseemann/prokka) [109] and RAST Rapid Annotation using Subsystem Technology (https://rast.nmpdr.org/) [110]. Ribosome RNA (rRNA) genes and transfer RNA (tRNA) genes were predicted using tRNAscan-SE galaxy version 0.4 [111]. The GEIs were predicted by IslandViewer 4 (http://www.pathogenomics.sfu.ca/islandviewer/upload/), which is an integrated interface for computational identification and visualization of genomic islands [112]. PHASTER (https://phaster.ca/) was used for prophage prediction [113] and the CRISPRCasFinder 1.1.2 (https://crisprcas.i2bc.paris-saclay.fr/) for the CRISPR identification. Gene functions were analyzed by BLASTP using Cluster of Orthologous Groups (COG) of proteins on WebMGA server [114], Kyoto Encyclopedia of Genes and Genomes (KEGG) [115], and CAZy enzymes using dbCAN2 (http://bcb.unl.edu/dbCAN2/index.php) [116]. The potential secondary metabolite biosynthetic gene clusters were investigated using antiSMASH v. 5.1.1 [117]. KEGG and COG annotations were carried out for proteins predicted with Prokka. dbCAN2 and antiSMASH were run with the genomic DNA sequence.

For a better understanding of the adaptation and survival strategies of the Saharan bacteria, two *Streptomyces* strains were selected from the database for comparison, *Streptomyces fildesensis* So13.3, (CP048835.1) with a linear genome of 9.47 Mb isolated from the Antarctic, and *Streptomyces* sp. strain Wb2n-11 (CVPB00000000) with a genome size of 8.23 Mb isolated from the Sinai desert in Egypt [44, 118]. The analysis and comparison between these *Streptomyces* strains was based on the secondary metabolites, Cluster of Orthologous Groups. A phylogenetic analysis employing concatenated sequences of the housekeeping genes *atpD* (ATP synthase F1, *β*-subunit), *gyrB* (DNA gyrase, B subunit), *recA* (recombinase A), *rpoB* (RNA polymerase, *β*-subunit) and *trpB* (tryptophan synthase, *β*-subunit) was carried out for all these strains, including six other desert species.

The DNA repair protein sequence of the genes labelled as *RuvA, RuvB, RuvC* and *RecG* was analyzed [119] using the database NCBI for strains *Streptomyces fildesensis* So13.3, (CP048835.1), *Streptomyces* sp. strain Wb2n-11 (CVPB00000000), *Actinopolyspora saharensis* DSM 45459 (NZ_FNKO00000000), *Actinopolyspora righensis* DSM 45501 (NZ_FPAT00000000.1), *Thermobifida halotolerans* DSM 44931 (NZ_JRUV01000210.1), *Geodermatophilus saharensis* DSM 45423 (NZ_FZOH01000001.1), *Saccharothrix saharensis* DSM 45456 (NZ_VFPP00000000.1). *Streptomyces* sp.DH-12 (NZ_PPFB00000000.1) were analyzed using Clustal W and Mega 7 software [106, 107].

## Supplementary Information

**Additional file 1: Figure S1.** Growth parameters of strains Babs14 and Osf17. Growth as a function of (**A**) pH, (**B**) temperature, and (**C**) salinity using NaCl. **Figure S2**. (**A**) Comparison of the genomes of *Streptomyces* sp. Babs14 (used as reference sequence) and *Streptomyces* sp. Osf17 using SEED Viewer version 2.0. The result lists the genes of the reference organism in chromosomal order and display hits on the comparison organism. (**B**) Color codes of comparison circles for Figs. S2, S3 and S4. **Figure S3.** Alignment of *Streptomyces* sp. Babs14 (used as reference sequence) and *Streptomyces* sp. SGAir 0924 (CP027297.1) using SEED Viewer version 2.0. **Figure S4**. Alignment of *Streptomyces* sp. Osf17 (used as reference sequence) and *Streptomyces* sp. SS52 (NZ_CP039123) using SEED Viewer version 2.0. **Figure S5:** Dot plot matches of *Streptomyces* sp. Babs14 with *Streptomyces* sp. Osf17 generated from SEED Viewer Version 2.0. **Figure S6:** Dot plot matches of *Streptomyces* sp. Babs14 with *Streptomyces* sp. SGAir0924 (CP027297.1) generated from SEED Viewer Version 2.0. **Figure S7:** Dot plot matches of *Streptomyces* sp. Osf17 with *Streptomyces* sp. SS52 (NZ_CP039123) generated from SEED Viewer Version 2.0. **Figure S8.** Subsystem feature distribution of the strains Babs14 (**A**) and Osf17 (**B**) using SEED Viewer version 2.0. on RAST subsystem technology. **Figure S9.** Assembly graph layout of *Streptomyces* sp. Babs14. **Figure S10.** Assembly graph layout of *Streptomyces* sp. Osf17. **Figure S11.** Cluster 21 predicted in *Streptomyces* sp. Babs14 using antiSMASH v. 5.1.1.

**Additional file 2: Table S1.** Biological processes of the 284 protein clusters shared by strains Babs14 and Osf17 as detected by the Orthovenn2 webserver. **Table S2.** Genomic islands of *Streptomyces* sp. Babs14 and *Streptomyces* sp. Osf17 detected using IslandViewer 4. **Table S3.** Prophage regions predicted in Babs14 and Osf17 using PHASTER. **Table S4**. Potential gene clusters that encode for secondary metabolites of Babs14 predicted by antiSMASH version 5.1.1. **Table S5.** Potential gene clusters that encode secondary metabolites of Osf17 predicted by antiSMASH version 5.1.1. **Table S6.** Stress response of (**A**) *Streptomyces* sp. Babs14, and (**B**) *Streptomyces* sp. Osf17, using the SEED Viewer version 2.0.

## Data Availability

All data generated or analyzed during this study are included in this published article [and its supplementary information files]. The genomic sequences described here have been submitted to NCBI GenBank under BioPoject ID PRJNA665615, and BioSample accessions SAMN16261965 and SAMN16261966. The genome sequences are available by searching the accessions JADBJQ000000000 and JADBJP000000000 on the NCBI Nucleotide database.

## Abbreviations

ANI: Average nucleotide identity
BGCs: Biosynthetic gene clusters
COG: Cluster of Orthologous Groups
Csp: Cold shock proteins
ECF: The extracytoplasmic function
GEIs: The genomic islands
Hsp: Heat shock protein
ORF: Open reading frame
PCD: Programmed Cell Death
TAs: Toxin-antitoxin systems
*AtpD*: ATP synthase F1, *β*-subunit
*GyrB*: DNA gyrase, B subunit
*RecA*: Recombinase A
*RpoB*: RNA polymerase, *β*-subunit
*TrpB*: Tryptophan synthase, *β*-subunit
