# Supplementary material for: Whole-genome sequencing of two Streptomyces strains isolated from the sand dunes of Sahara
Source: BMC Genomics. 2021 Jul 27;22:578. doi: 10.1186/s12864-021-07866-x (PMC8317367; doi:10.1186/s12864-021-07866-x)
Supplement: Supplementary file 1 — Additional file 1: Figure S1. Growth parameters of strains Babs14 and Osf17. Growth as a function of (A) pH, (B) temperature, and (C) salinity using NaCl. Figure S2. (A) Comparison of the genomes of Streptomyces sp. Babs14 (used as reference sequence) and Streptomyces sp. Osf17 using SEED Viewer version 2.0. The result lists the genes of the reference organism in chromosomal order and display hits on the comparison organism. (B) Color codes of comparison circles for Figs. S2, S3 and S4. Figure S3. Alignment of Streptomyces sp. Babs14 (used as reference sequence) and Streptomyces sp. SGAir 0924 (CP027297.1) using SEED Viewer version 2.0. Figure S4. Alignment of Streptomyces sp. Osf17 (used as reference sequence) and Streptomyces sp. SS52 (NZ_CP039123) using SEED Viewer version 2.0. Figure S5: Dot plot matches of Streptomyces sp. Babs14 with Streptomyces sp. Osf17 generated from SEED Viewer Version 2.0. Figure S6: Dot plot matches of Streptomyces sp. Babs14 with Streptomyces sp. SGAir0924 (CP027297.1) generated from SEED Viewer Version 2.0. Figure S7: Dot plot matches of Streptomyces sp. Osf17 with Streptomyces sp. SS52 (NZ_CP039123) generated from SEED Viewer Version 2.0. Figure S8. Subsystem feature distribution of the strains Babs14 (A) and Osf17 (B) using SEED Viewer version 2.0. on RAST subsystem technology. Figure S9. Assembly graph layout of Streptomyces sp. Babs14. Figure S10. Assembly graph layout of Streptomyces sp. Osf17. Figure S11. Cluster 21 predicted in Streptomyces sp. Babs14 using antiSMASH v. 5.1.1. [file 12864_2021_7866_MOESM1_ESM.docx]

***Additional file 1.***


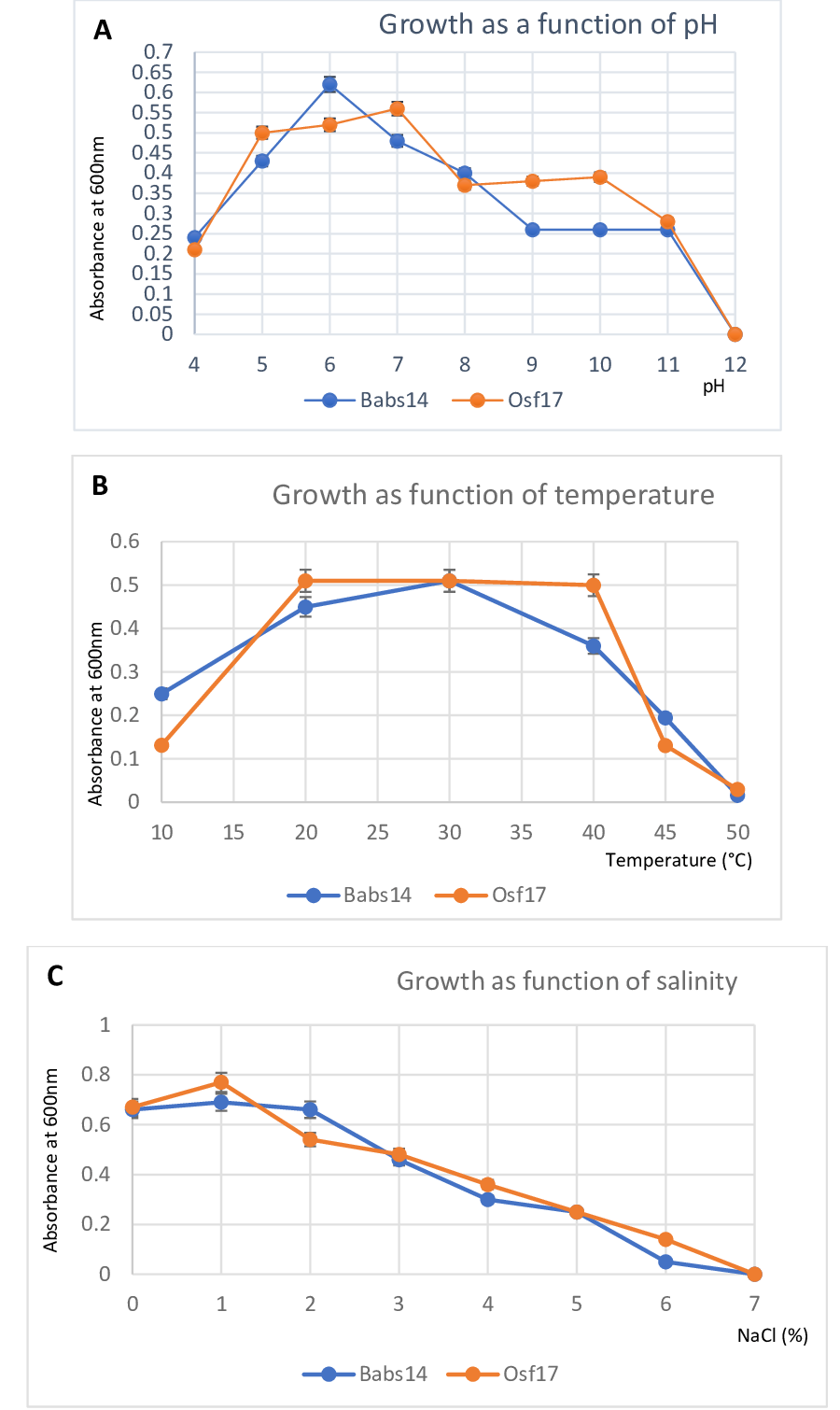


**Figure S1.** Growth parameters of strains Babs14 and Osf17. Growth as a function of **(A)** pH, **(B)** temperature, and **(C)** salinity using NaCl.

**A**


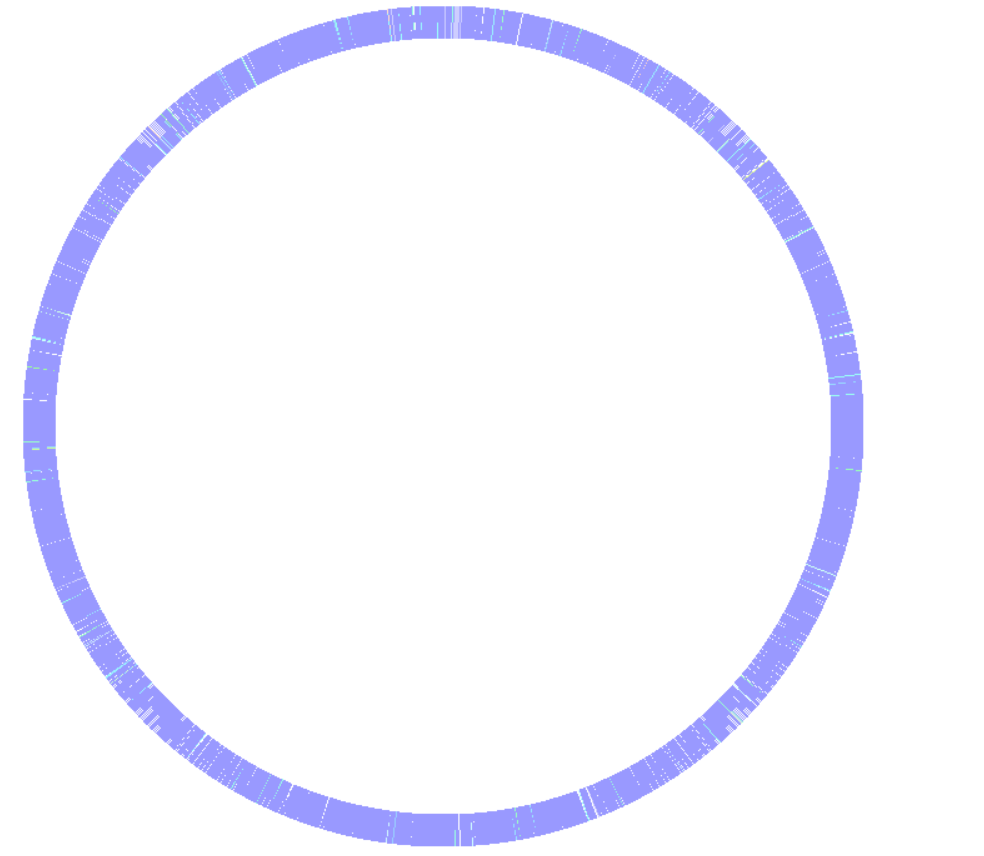


**B**


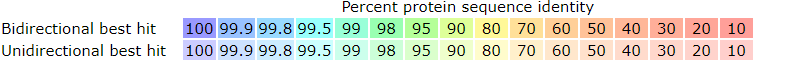


**Figure S2**. **(A)** Comparison of the genomes of *Streptomyces* sp. Babs14 (used as reference sequence) and *Streptomyces* sp. Osf17 using SEED Viewer version 2.0. The result lists the genes of the reference organism in chromosomal order and display hits on the comparison organism. **(B)** Color codes of comparison circles for Figs. S2, S3 and S4.


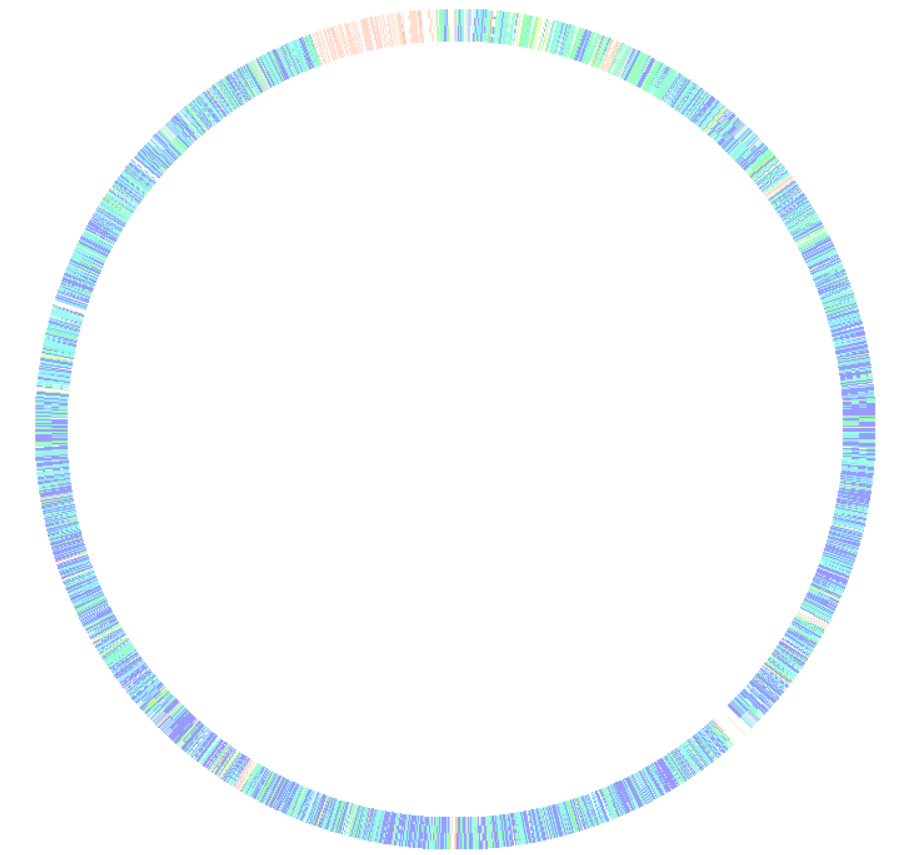


**Figure S3.** Alignment of *Streptomyces* sp. Babs14 (used as reference sequence) and *Streptomyces* sp. SGAir 0924 (CP027297.1) using SEED Viewer version 2.0.


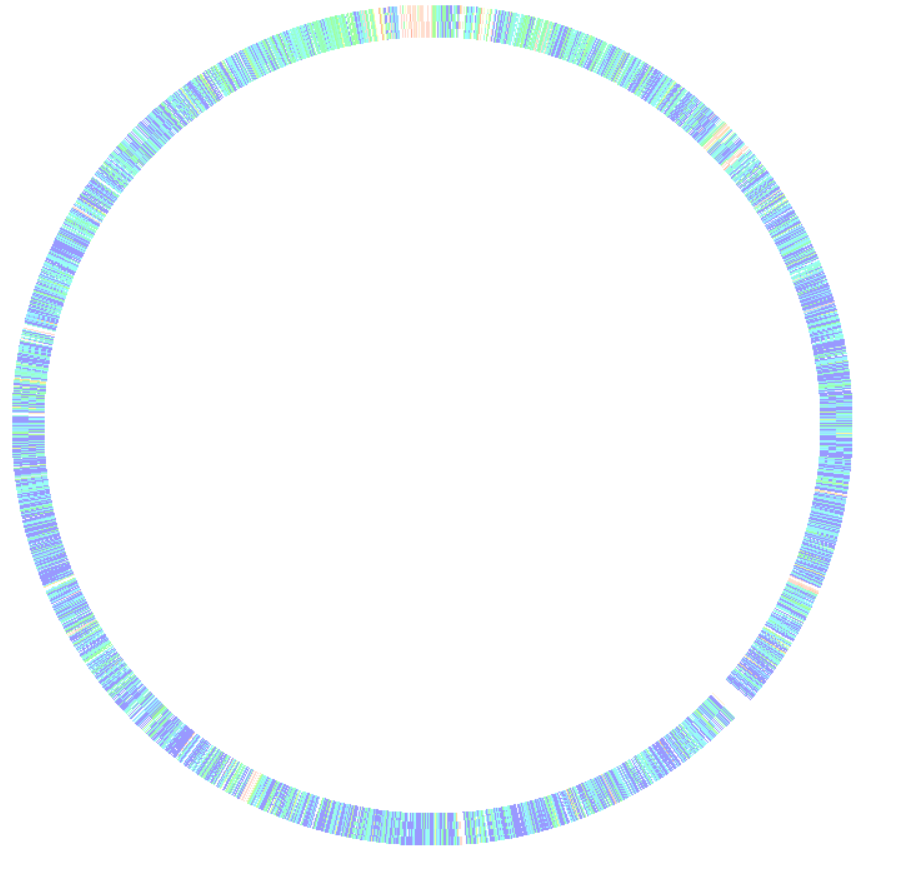


**Figure S4**. Alignment of *Streptomyces* sp. Osf17 (used as reference sequence) and *Streptomyces* sp. SS52 (NZ_CP039123) using SEED Viewer version 2.0.


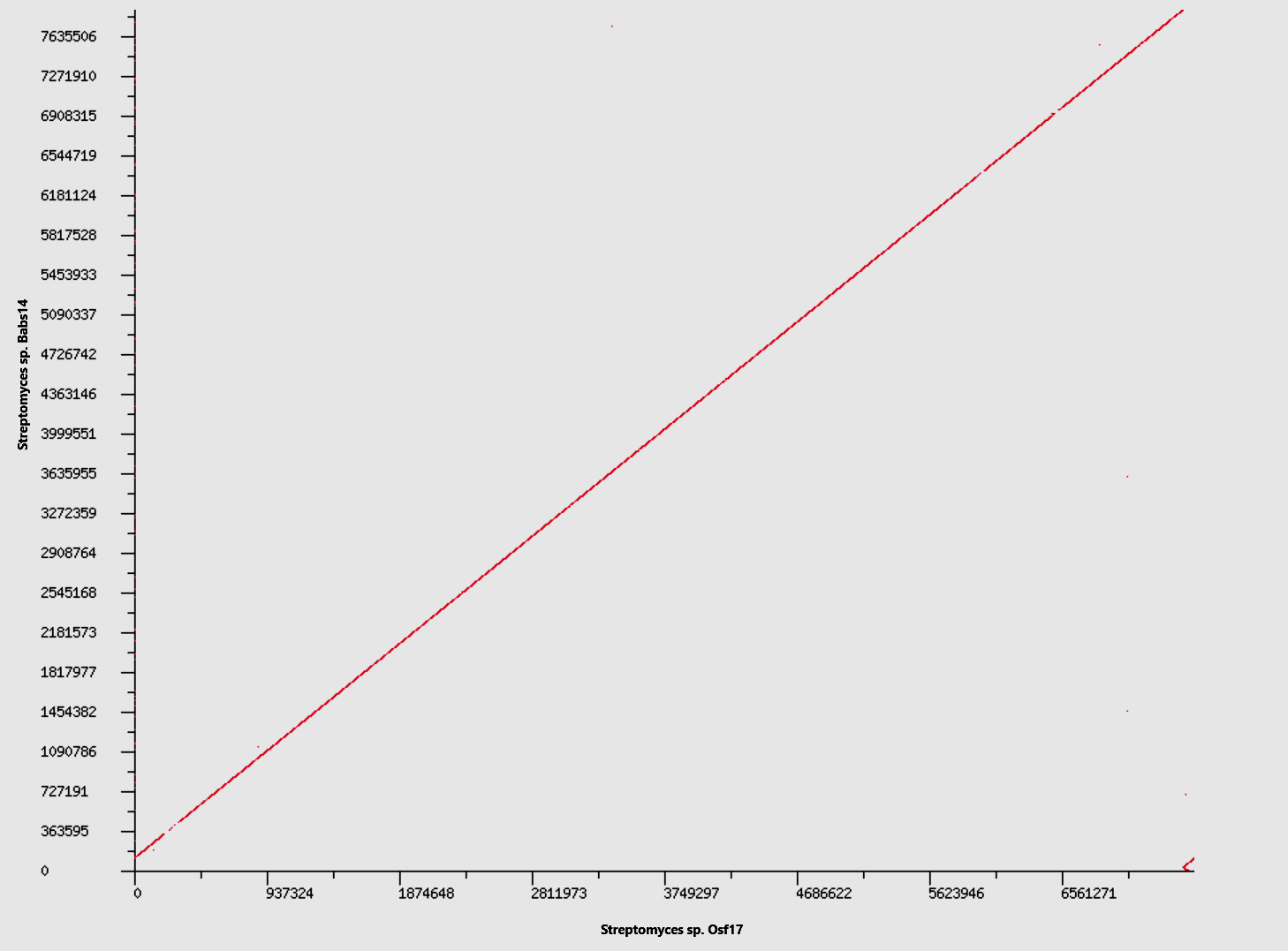


**Figure S5:** Dot plot matches of *Streptomyces* sp. Babs14 with *Streptomyces* sp. Osf17 generated from SEED Viewer Version 2.0.


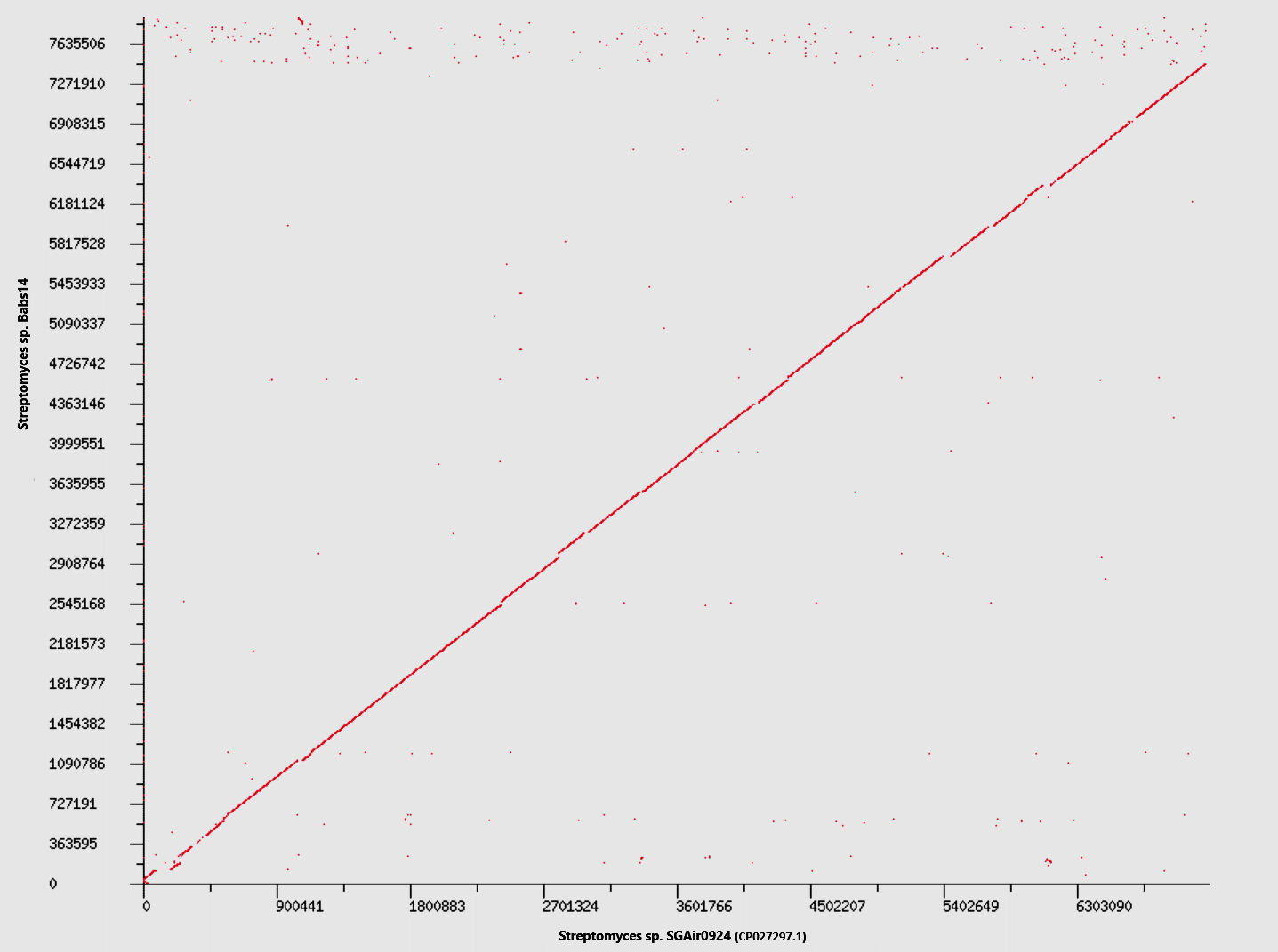


**Figure S6:** Dot plot matches of *Streptomyces* sp. Babs14 with *Streptomyces* sp. SGAir0924 (CP027297.1) generated from SEED Viewer Version 2.0.


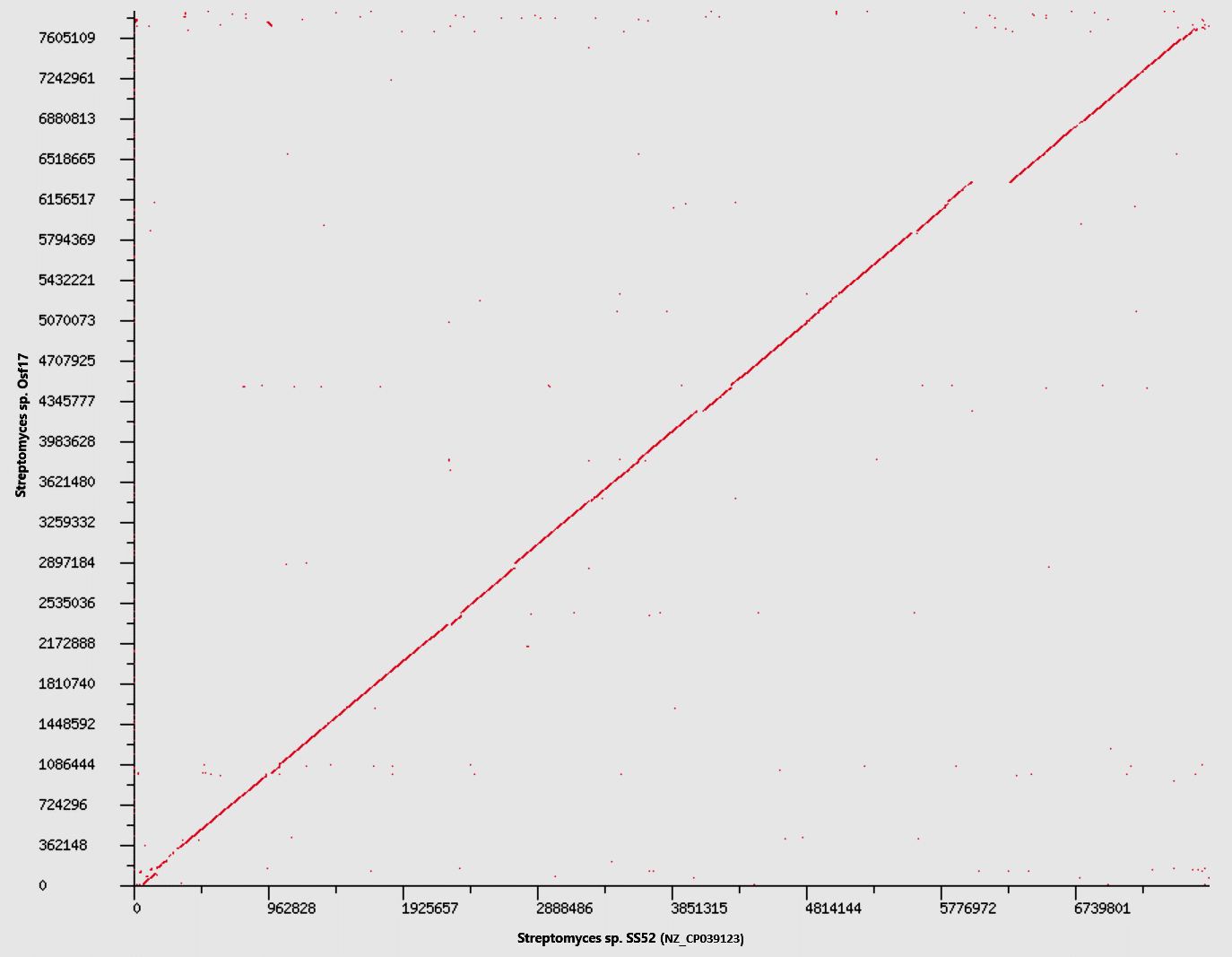


**Figure S7:** Dot plot matches of *Streptomyces* sp. Osf17 with *Streptomyces* sp. SS52 (NZ_CP039123) generated from SEED Viewer Version 2.0.

**A**


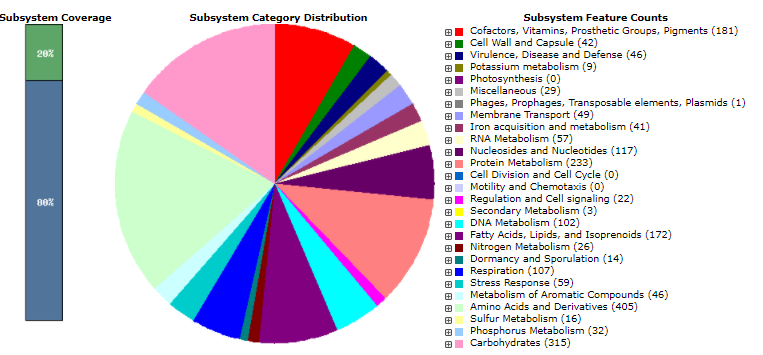


**B**


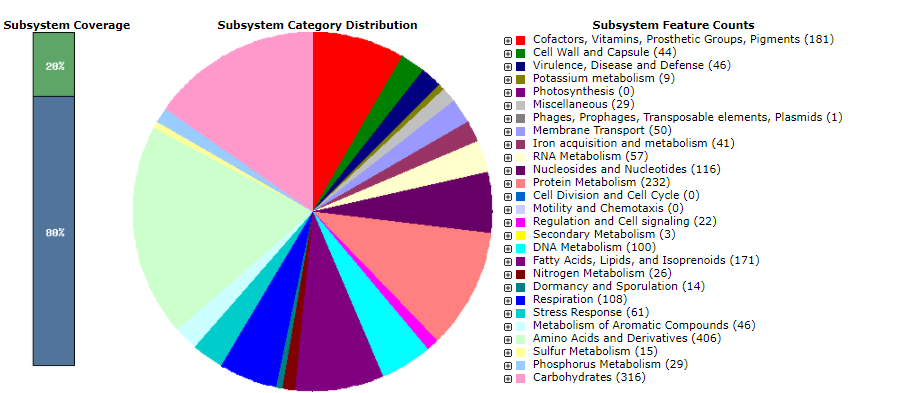


**Figure S8.** Subsystem feature distribution of the strains Babs14 **(A)** and Osf17 **(B)** using SEED Viewer version 2.0. on RAST subsystem technology.


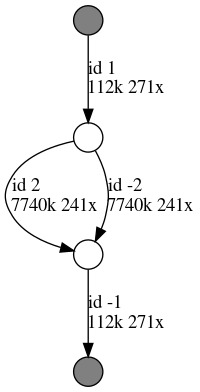


**Figure S9.** Assembly graph layout of *Streptomyces* sp. Babs14.


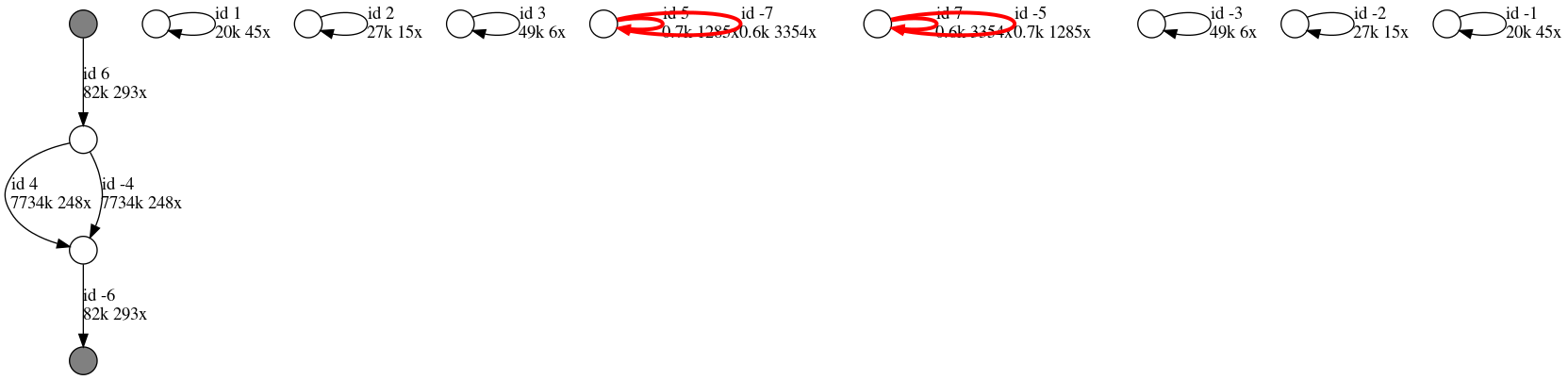


**Figure S10.** Assembly graph layout of *Streptomyces* sp. Osf17.


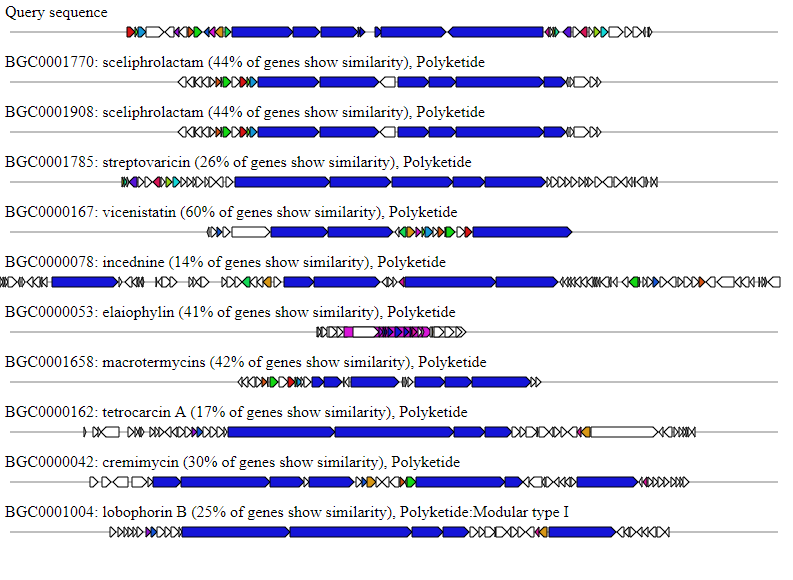


**Figure S11.** The similar BGC clusters detected for cluster 21 of *Streptomyces* sp. Babs14 by using antiSMASH v. 5.1.1.
