# Supplementary material for: Whole-genome sequencing of two Streptomyces strains isolated from the sand dunes of Sahara
Source: BMC Genomics. 2021 Jul 27;22:578. doi: 10.1186/s12864-021-07866-x (PMC8317367; doi:10.1186/s12864-021-07866-x)
Supplement: Supplementary file 2 — Additional file 2: Table S1. Biological processes of the 284 protein clusters shared by strains Babs14 and Osf17 as detected by the Orthovenn2 webserver. Table S2. Genomic islands of Streptomyces sp. Babs14 and Streptomyces sp. Osf17 detected using IslandViewer 4. Table S3. Prophage regions predicted in Babs14 and Osf17 using PHASTER. Table S4. Potential gene clusters that encode for secondary metabolites of Babs14 predicted by antiSMASH version 5.1.1. Table S5. Potential gene clusters that encode secondary metabolites of Osf17 predicted by antiSMASH version 5.1.1. Table S6. Stress response of (A) Streptomyces sp. Babs14, and (B) Streptomyces sp. Osf17, using the SEED Viewer version 2.0. [file 12864_2021_7866_MOESM2_ESM.docx]

***Additional file 2.***

| Table S1. Biological processes of the 284 protein clusters shared by strains Babs14 and Osf17 as detected by the Orthovenn2 webserver. | |
| --- | --- |
| Biological functions | Amount of protein clusters |
| carbohydrate metabolic process | 5 |
| polysaccharide metabolic process | 1 |
| organic acid metabolic process | 5 |
| generation of precursor metabolites and energy | 1 |
| nucleobase-containing compound metabolic process | 12 |
| DNA metabolic process | 4 |
| DNA modification | 1 |
| protein folding | 1 |
| cellular protein modification process | 1 |
| lipid metabolic process | 3 |
| cellular aromatic compound metabolic process | 12 |
| one-carbon metabolic process | 1 |
| phosphorus metabolic process | 3 |
| nitrogen compound metabolic process | 15 |
| transport | 1 |
| biological_process | 22 |
| metabolic process | 21 |
| nucleoside metabolic process | 1 |
| nucleotide metabolic process | 3 |
| nucleotide-sugar metabolic process | 1 |
| genetic transfer | 1 |
| amine metabolic process | 1 |
| cellular process | 10 |
| cellular component organization | 1 |
| RNA metabolic process | 3 |
| transposition | 3 |
| developmental process | 1 |
| cellular component morphogenesis | 1 |
| macromolecule metabolic process | 8 |
| cellular metabolic process | 18 |
| primary metabolic process | 14 |
| cellular lipid metabolic process | 1 |
| heterocycle metabolic process | 12 |
| response to stimulus | 3 |
| cofactor metabolic process | 2 |
| establishment of localization | 1 |
| biological regulation | 4 |
| nucleobase-containing compound metabolic process | 1 |
| cellular protein modification process | 1 |
| cellular aromatic compound metabolic process | 1 |
| phosphorus metabolic process | 1 |
| nitrogen compound metabolic process | 1 |
| biological_process | 3 |
| metabolic process | 2 |
| cellular process | 2 |
| RNA metabolic process | 1 |
| protein metabolic process | 1 |
| macromolecule metabolic process | 2 |
| cellular metabolic process | 2 |
| primary metabolic process | 2 |
| heterocycle metabolic process | 1 |
| response to stimulus | 1 |
| biological regulation | 2 |
| nucleobase-containing compound metabolic process | 1 |
| DNA modification | 1 |
| cellular aromatic compound metabolic process | 1 |
| one-carbon metabolic process | 1 |
| vitamin metabolic process | 1 |
| nitrogen compound metabolic process | 2 |
| transport | 1 |
| biological_process | 3 |
| metabolic process | 2 |
| cellular process | 2 |
| RNA metabolic process | 1 |
| macromolecule metabolic process | 1 |
| cellular metabolic process | 2 |
| primary metabolic process | 1 |
| heterocycle metabolic process | 2 |
| response to stimulus | 1 |
| establishment of localization | 1 |
| biological regulation | 1 |

| Table S2. Genomic islands of *Streptomyces* sp. Babs14 and Osf17 detected using IslandViewer 4. | | |
| --- | --- | --- |
| GEIs | *GEI size of Streptomyces* sp. Babs14 | *GEI size of*  *Streptomyces* sp. Osf17 |
| GEI 1 | 28,653 | 9,957 |
| GEI 2 | 12,948 | 11,923 |
| GEI 3 | 11,616 | 11,616 |
| GEI 4 | 14,608 | 14,608 |
| GEI 5 | 10,214 | 10,214 |
| GEI 6 | 12,055 | 9,419 |
| GEI 7 | 18,911 | 18,911 |
| GEI 8 | 9,463 | 8,501 |
| GEI 9 | 6,32 | 22,316 |
| GEI 10 | 9,694 | 6,32 |
| GEI 11 | 31,354 | 9,694 |
| GEI 12 | 38,782 | 31,354 |
| GEI 13 | - | 65,802 |

| Table S3. Prophage regions predicted in Babs14 and Osf17 using PHASTER. | | | | | | | | | |
| --- | --- | --- | --- | --- | --- | --- | --- | --- | --- |
| Strains | Region length | Completeness | Score (%) | Amount of CDSs | Region position | Possible phage | | GC % | Accession |
| Babs14 | 10.9 Kb | incomplete | 40 | 17 | 2906194-2917100 | Phage Rhodococcus REQ1 | 72.40 | | NC_016655 |
| Osf17 | 8.4 Kb | incomplete | 30 | 9 | 1870238-1878727 | Phage Bacillus G | 72.43 | | NC_023719 |
| Osf17 | 25.5 Kb | incomplete | 30 | 36 | 2905990-2931526 | Phage Gordonia Nymphadora | 71.78 | | NC_031061 |
| Osf17 | 8.6 Kb | incomplete | 30 | 10 | 6774978-6783629 | Phage Erwinia vB EamM EarlPhillipI | 73.93 | | NC_031007 |

| **Table S4**. Potential gene clusters that encode for secondary metabolites of Babs14 predicted by antiSMASH version 5.1.1. | | | | | | | |
| --- | --- | --- | --- | --- | --- | --- | --- |
| **Region** | **Type** | **From** | **To** | **Most similar known cluster** | **Similarity**  **%** | | |
| Cluster 1C | [terpene](https://docs.antismash.secondarymetabolites.org/glossary/#terpene) | 18,337 | 42,741 | [isorenieratene](https://mibig.secondarymetabolites.org/go/BGC0000664/1) |  | 100 |  |
| Cluster 2 | [NRPS](https://docs.antismash.secondarymetabolites.org/glossary/#nrps),[T1PKS](https://docs.antismash.secondarymetabolites.org/glossary/#t1pks),[NRPS-like](https://docs.antismash.secondarymetabolites.org/glossary/#nrps-like),[hglE-KS](https://docs.antismash.secondarymetabolites.org/glossary/#hgle-ks) | 159,773 | 369,779 | [candicidin](https://mibig.secondarymetabolites.org/go/BGC0000034/1) |  | 95 |  |
| Cluster 3 | [terpene](https://docs.antismash.secondarymetabolites.org/glossary/#terpene) | 388,546 | 409,134 | [lysolipin I](https://mibig.secondarymetabolites.org/go/BGC0000242/1) |  | 4 |  |
| Cluster 4 | [indole](https://docs.antismash.secondarymetabolites.org/glossary/#indole) | 713,952 | 735,073 | [5-isoprenylindole-3-carboxylate β-D-glycosyl ester](https://mibig.secondarymetabolites.org/go/BGC0001483/1) |  | 33 |  |
| Cluster 5 | [terpene](https://docs.antismash.secondarymetabolites.org/glossary/#terpene) | 793,773 | 817,176 | [carotenoid](https://mibig.secondarymetabolites.org/go/BGC0000633/1) |  | 54 |  |
| Cluster 6 | [T3PKS](https://docs.antismash.secondarymetabolites.org/glossary/#t3pks) | 1,284,413 | 1,325,519 | [herboxidiene](https://mibig.secondarymetabolites.org/go/BGC0001065/1) |  | 8 |  |
| Cluster 7 | [ectoine](https://docs.antismash.secondarymetabolites.org/glossary/#ectoine) | 1,985,299 | 1,995,697 | [ectoine](https://mibig.secondarymetabolites.org/go/BGC0000853/1) |  | 100 |  |
| Cluster 8 | [melanin](https://docs.antismash.secondarymetabolites.org/glossary/#melanin) | 2,930,499 | 2,941,107 | [melanin](https://mibig.secondarymetabolites.org/go/BGC0000909/1) |  | 60 |  |
| Cluster 9 | [siderophore](https://docs.antismash.secondarymetabolites.org/glossary/#siderophore) | 3,027,662 | 3,038,619 | [desferrioxamin B / desferrioxamine E](https://mibig.secondarymetabolites.org/go/BGC0000940/1) |  | 83 |  |
| Cluster 10 | [NRPS-like](https://docs.antismash.secondarymetabolites.org/glossary/#nrps-like) | 4,035,802 | 4,078,834 | [alanylclavam / 2-hydroxymethylclavam / 2-formyloxymethylclavam / clavam-2-carboxylate](https://mibig.secondarymetabolites.org/go/BGC0000843/1) |  | 12 |  |
| Cluster 11 | [lanthipeptide](https://docs.antismash.secondarymetabolites.org/glossary/#lanthipeptide) | 4,297,384 | 4,318,612 | [catenulipeptin](https://mibig.secondarymetabolites.org/go/BGC0000501/1) |  | 60 |  |
| Cluster 12 | [NRPS](https://docs.antismash.secondarymetabolites.org/glossary/#nrps) | 4,545,665 | 4,608,045 | [ansamitocin P-3](https://mibig.secondarymetabolites.org/go/BGC0001511/1) |  | 7 |  |
| Cluster 13 | [terpene](https://docs.antismash.secondarymetabolites.org/glossary/#terpene) | 5,271,650 | 5,292,271 | [albaflavenone](https://mibig.secondarymetabolites.org/go/BGC0000660/1) |  | 100 |  |
| Cluster 14 | [T2PKS](https://docs.antismash.secondarymetabolites.org/glossary/#t2pks) | 5,335,903 | 5,408,499 | [spore pigment](https://mibig.secondarymetabolites.org/go/BGC0000271/1) |  | 66 |  |
| Cluster 15 | [siderophore](https://docs.antismash.secondarymetabolites.org/glossary/#siderophore) | 5,851,047 | 5,861,175 | - | | | |
| Cluster 16 | [bacteriocin](https://docs.antismash.secondarymetabolites.org/glossary/#bacteriocin) | 6,095,284 | 6,105,691 | - | | | |
| Cluster 17 | [terpene](https://docs.antismash.secondarymetabolites.org/glossary/#terpene) | 6,121,923 | 6,141,888 | [geosmin](https://mibig.secondarymetabolites.org/go/BGC0001181/1) |  | 100 |  |
| Cluster 18 | [siderophore](https://docs.antismash.secondarymetabolites.org/glossary/#siderophore) | 6,295,834 | 6,309,007 | [paulomycin](https://mibig.secondarymetabolites.org/go/BGC0001732/1) |  | 9 |  |
| Cluster 19 | [NRPS](https://docs.antismash.secondarymetabolites.org/glossary/#nrps) | 6,325,885 | 6,404,464 | [CDA1b / CDA2a / CDA2b / CDA3a / CDA3b / CDA4a / CDA4b](https://mibig.secondarymetabolites.org/go/BGC0000315/1) |  | 72 |  |
| Cluster 20 | [terpene](https://docs.antismash.secondarymetabolites.org/glossary/#terpene) | 6,738,819 | 6,764,741 | [hopene](https://mibig.secondarymetabolites.org/go/BGC0000663/1) |  | 100 |  |
| Cluster 21 | [T1PKS](https://docs.antismash.secondarymetabolites.org/glossary/#t1pks) | 6,874,946 | 6,969,936 | sceliphrolactam |  | 44 |  |
| Cluster 22 | [terpene](https://docs.antismash.secondarymetabolites.org/glossary/#terpene) | 7,097,617 | 7,118,648 | [versipelostatin](https://mibig.secondarymetabolites.org/go/BGC0001204/1) |  | 5 |  |
| Cluster 23 | [bacteriocin](https://docs.antismash.secondarymetabolites.org/glossary/#bacteriocin) | 7,131,633 | 7,141,848 | [informatipeptin](https://mibig.secondarymetabolites.org/go/BGC0000518/1) |  | 42 |  |
| Cluster 24 | [NRPS](https://docs.antismash.secondarymetabolites.org/glossary/#nrps) | 7,401,716 | 7,452,300 | [coelichelin](https://mibig.secondarymetabolites.org/go/BGC0000325/1) |  | 100 |  |
| Cluster 25 | [NRPS](https://docs.antismash.secondarymetabolites.org/glossary/#nrps) | 7,545,386 | 7,597,798 | [coelibactin](https://mibig.secondarymetabolites.org/go/BGC0000324/1) |  | 100 |  |
| Cluster 26 | [lanthipeptide](https://docs.antismash.secondarymetabolites.org/glossary/#lanthipeptide) | 7,597,857 | 7,620,346 | - | | | |
| Cluster 27 | [lanthipeptide](https://docs.antismash.secondarymetabolites.org/glossary/#lanthipeptide) | 7,632,588 | 7,655,170 | [SapB](https://mibig.secondarymetabolites.org/go/BGC0000551/1) |  | 100 |  |
| Cluster 28 | [NRPS](https://docs.antismash.secondarymetabolites.org/glossary/#nrps) | 7,822,785 | 7,884,453 | [streptothricin](https://mibig.secondarymetabolites.org/go/BGC0000432/1) |  | 91 |  |
| Cluster 29 | T2PKS, butyrolactone | 53,536 | 114,649 | fluostatins M-Q |  | 67 |  |

| **Table S5.** Potential gene clusters that encode secondary metabolites of Osf17 predicted by antiSMASH version 5.1.1. | | | | | | | |
| --- | --- | --- | --- | --- | --- | --- | --- |
| **Clusters** | **Type** | **From** | | **To** | **Most similar known cluster** | **Similarity (%)** | |
| Cluster1C | [terpene](https://docs.antismash.secondarymetabolites.org/glossary/#terpene) | 18,069 | | 42,739 | [isorenieratene](https://mibig.secondarymetabolites.org/go/BGC0000664/1) |  | 100 |
| Cluster 2 | [NRPS](https://docs.antismash.secondarymetabolites.org/glossary/#nrps),[T1PKS](https://docs.antismash.secondarymetabolites.org/glossary/#t1pks),[NRPS-like](https://docs.antismash.secondarymetabolites.org/glossary/#nrps-like),[hglE-KS](https://docs.antismash.secondarymetabolites.org/glossary/#hgle-ks) | 159,771 | | 369,557 | [levorin A3 / C06690 / FR-008-III / candicidin A / UNII-AP5PEF5W7U](https://mibig.secondarymetabolites.org/go/BGC0000061/1) |  | 95 |
| Cluster 3 | [terpene](https://docs.antismash.secondarymetabolites.org/glossary/#terpene) | 388,316 | | 408,904 | [lysolipin I](https://mibig.secondarymetabolites.org/go/BGC0000242/1) |  | 4 |
| Cluster 4 | [indole](https://docs.antismash.secondarymetabolites.org/glossary/#indole) | 713,719 | | 734,840 | [5-isoprenylindole-3-carboxylate β-D-glycosyl ester](https://mibig.secondarymetabolites.org/go/BGC0001483/1) |  | 33 |
| Cluster 5 | [terpene](https://docs.antismash.secondarymetabolites.org/glossary/#terpene) | 793,540 | | 816,943 | [carotenoid](https://mibig.secondarymetabolites.org/go/BGC0000633/1) |  | 54 |
| Cluster 6 | [T3PKS](https://docs.antismash.secondarymetabolites.org/glossary/#t3pks) | 1,284,163 | | 1,325,269 | [herboxidiene](https://mibig.secondarymetabolites.org/go/BGC0001065/1) |  | 8 |
| Cluster 7 | [ectoine](https://docs.antismash.secondarymetabolites.org/glossary/#ectoine) | 1,985,056 | | 1,995,454 | [ectoine](https://mibig.secondarymetabolites.org/go/BGC0000853/1) |  | 100 |
| Cluster 8 | [melanin](https://docs.antismash.secondarymetabolites.org/glossary/#melanin) | 2,930,295 | | 2,940,753 | [melanin](https://mibig.secondarymetabolites.org/go/BGC0000909/1) |  | 60 |
| Cluster 9 | [siderophore](https://docs.antismash.secondarymetabolites.org/glossary/#siderophore) | 3,027,458 | | 3,038,415 | [desferrioxamin B / desferrioxamine E](https://mibig.secondarymetabolites.org/go/BGC0000940/1) |  | 83 |
| Cluster 10 | [NRPS-like](https://docs.antismash.secondarymetabolites.org/glossary/#nrps-like) | 4,035,113 | | 4,078,145 | [alanylclavam / 2-hydroxymethylclavam / 2-formyloxymethylclavam / clavam-2-carboxylate](https://mibig.secondarymetabolites.org/go/BGC0000843/1) |  | 12 |
| Cluster 11 | [lanthipeptide](https://docs.antismash.secondarymetabolites.org/glossary/#lanthipeptide) | 4,296,694 | | 4,317,922 | [catenulipeptin](https://mibig.secondarymetabolites.org/go/BGC0000501/1) |  | 60 |
| Cluster 12 | [NRPS](https://docs.antismash.secondarymetabolites.org/glossary/#nrps) | 4,544,782 | | 4,607,348 | [ansamitocin P-3](https://mibig.secondarymetabolites.org/go/BGC0001511/1) |  | 7 |
| Cluster 13 | [terpene](https://docs.antismash.secondarymetabolites.org/glossary/#terpene) | 5,270,962 | | 5,291,583 | [albaflavenone](https://mibig.secondarymetabolites.org/go/BGC0000660/1) |  | 100 |
| Cluster 14 | [T2PKS](https://docs.antismash.secondarymetabolites.org/glossary/#t2pks) | 5,335,216 | | 5,407,812 | [spore pigment](https://mibig.secondarymetabolites.org/go/BGC0000271/1) |  | 66 |
| Cluster 15 | [siderophore](https://docs.antismash.secondarymetabolites.org/glossary/#siderophore) | 5,850,347 | | 5,860,474 |  | | |
| Cluster 16 | [bacteriocin](https://docs.antismash.secondarymetabolites.org/glossary/#bacteriocin) | 6,094,576 | | 6,104,985 |  | | |
| Cluster 17 | [terpene](https://docs.antismash.secondarymetabolites.org/glossary/#terpene) | 6,121,214 | | 6,141,179 | [geosmin](https://mibig.secondarymetabolites.org/go/BGC0001181/1) |  | 100 |
| Cluster 18 | [siderophore](https://docs.antismash.secondarymetabolites.org/glossary/#siderophore) | 6,295,129 | | 6,308,302 | [paulomycin](https://mibig.secondarymetabolites.org/go/BGC0001732/1) |  | 9 |
| Cluster 19 | [NRPS](https://docs.antismash.secondarymetabolites.org/glossary/#nrps) | 6,325,180 | | 6,403,758 | [CDA1b / CDA2a / CDA2b / CDA3a / CDA3b / CDA4a / CDA4b](https://mibig.secondarymetabolites.org/go/BGC0000315/1) |  | 72 |
| Cluster 20 | [terpene](https://docs.antismash.secondarymetabolites.org/glossary/#terpene) | 6,738,113 | | 6,764,035 | [hopene](https://mibig.secondarymetabolites.org/go/BGC0000663/1) |  | 100 |
| Cluster 21 | [T1PKS](https://docs.antismash.secondarymetabolites.org/glossary/#t1pks) | 6,874,238 | | 6,969,171 | [streptovaricin](https://mibig.secondarymetabolites.org/go/BGC0001785/1) |  | 31 |
| Cluster 22 | [terpene](https://docs.antismash.secondarymetabolites.org/glossary/#terpene) | 7,096,916 | | 7,117,947 | [versipelostatin](https://mibig.secondarymetabolites.org/go/BGC0001204/1) |  | 5 |
| Cluster 23 | [bacteriocin](https://docs.antismash.secondarymetabolites.org/glossary/#bacteriocin) | 7,130,932 | | 7,141,147 | [informatipeptin](https://mibig.secondarymetabolites.org/go/BGC0000518/1) |  | 42 |
| Cluster 24 | [NRPS](https://docs.antismash.secondarymetabolites.org/glossary/#nrps) | 7,401,011 | | 7,451,595 | [coelichelin](https://mibig.secondarymetabolites.org/go/BGC0000325/1) |  | 100 |
| Cluster 25 | [NRPS](https://docs.antismash.secondarymetabolites.org/glossary/#nrps) | 7,544,681 | | 7,597,089 | [coelibactin](https://mibig.secondarymetabolites.org/go/BGC0000324/1) |  | 100 |
| Cluster 26 | [lanthipeptide](https://docs.antismash.secondarymetabolites.org/glossary/#lanthipeptide) | 7,597,148 | | 7,619,637 |  |  | |
| Cluster 27 | [lanthipeptide](https://docs.antismash.secondarymetabolites.org/glossary/#lanthipeptide) | 7,631,879 | | 7,654,461 | [SapB](https://mibig.secondarymetabolites.org/go/BGC0000551/1) |  | 100 |
| Cluer 28 | [NRPS](https://docs.antismash.secondarymetabolites.org/glossary/#nrps) | 7,822,216 | | 7,883,883 | [streptothricin](https://mibig.secondarymetabolites.org/go/BGC0000432/1) |  | 87 |
| Cluster 29 | [T2PKS](https://docs.antismash.secondarymetabolites.org/glossary/#t2pks),[butyrolactone](https://docs.antismash.secondarymetabolites.org/glossary/#butyrolactone) | | 22,257 | 83,375 | [fluostatins M-Q](https://mibig.secondarymetabolites.org/go/BGC0001596/1) | % | 67 |

|  |
| --- |
| \|  \| \| --- \| |
|  |
| **Table S6.** Stress response of **(A)** *Streptomyces* sp. Babs14, and **(B)** *Streptomyces* sp. Osf17, using the SEED Viewer version 2.0. |
| \| Presence \| Category \| Subcategory \| subsystem \| Proteins \| Active For A \| Active for B \| \| --- \| --- \| --- \| --- \| --- \| --- \| --- \| \| A and B \| Stress Response \| Detoxification \| Uptake of selenate and selenite \| DedA protein \| yes \| yes \| \| A and B \| Stress Response \| Osmotic stress \| Choline and Betaine Uptake and Betaine Biosynthesis \| Betaine aldehyde dehydrogenase (EC 1.2.1.8) \| yes \| yes \| \| A and B \| Stress Response \| Osmotic stress \| Choline and Betaine Uptake and Betaine Biosynthesis \| Glycine betaine ABC transport system, ATP-binding protein OpuAA (EC 3.6.3.32) \| yes \| yes \| \| A and B \| Stress Response \| Osmotic stress \| Choline and Betaine Uptake and Betaine Biosynthesis \| Glycine betaine ABC transport system, glycine betaine-binding protein OpuAC \| yes \| yes \| \| A and B \| Stress Response \| Osmotic stress \| Choline and Betaine Uptake and Betaine Biosynthesis \| Glycine betaine ABC transport system, permease protein OpuAB \| yes \| yes \| \| A and B \| Stress Response \| Osmotic stress \| Choline and Betaine Uptake and Betaine Biosynthesis \| Glycine betaine transporter OpuD \| yes \| yes \| \| A and B \| Stress Response \| Osmotic stress \| Osmoregulation \| Glycerol uptake facilitator protein \| yes \| yes \| \| A and B \| Stress Response \| Osmotic stress \| Osmoregulation \| Outer membrane protein A precursor \| yes \| yes \| \| A and B \| Stress Response \| Oxidative stress \| Glutathione: Biosynthesis and gamma-glutamyl cycle \| Gamma-glutamyltranspeptidase (EC 2.3.2.2) \| yes \| yes \| \| A and B \| Stress Response \| Oxidative stress \| Glutathione: Biosynthesis and gamma-glutamyl cycle \| Glutamate--cysteine ligase (EC 6.3.2.2) \| yes \| yes \| \| A and B \| Stress Response \| Oxidative stress \| Glutathione: Redox cycle \| Glutathione peroxidase (EC 1.11.1.9) \| yes \| yes \| \| A and B \| Stress Response \| Oxidative stress \| Glutathionylspermidine and Trypanothione \| Similarity with glutathionylspermidine synthase (EC 6.3.1.8), group 1 \| yes \| yes \| \| A and B \| Stress Response \| Oxidative stress \| Oxidative stress \| Alkyl hydroperoxide reductase subunit C-like protein \| yes \| yes \| \| A and B \| Stress Response \| Oxidative stress \| Oxidative stress \| Ferric uptake regulation protein FUR \| yes \| yes \| \| A and B \| Stress Response \| Oxidative stress \| Oxidative stress \| Nitrite-sensitive transcriptional repressor NsrR \| yes \| yes \| \| A and B \| Stress Response \| Oxidative stress \| Oxidative stress \| Organic hydroperoxide resistance protein \| yes \| yes \| \| A and B \| Stress Response \| Oxidative stress \| Oxidative stress \| Organic hydroperoxide resistance transcriptional regulator \| yes \| yes \| \| A and B \| Stress Response \| Oxidative stress \| Oxidative stress \| Redox-sensitive transcriptional activator SoxR \| yes \| yes \| \| A and B \| Stress Response \| Periplasmic Stress \| Periplasmic Stress Response \| Intramembrane protease RasP/YluC, implicated in cell division based on FtsL cleavage \| yes \| yes \| \| A and B \| Stress Response \| no subcategory \| Bacterial hemoglobins \| Hemoglobin-like protein HbO \| yes \| yes \| \| A and B \| Stress Response \| no subcategory \| Dimethylarginine metabolism \| NG,NG-dimethylarginine dimethylaminohydrolase 1 (EC 3.5.3.18) \| yes \| yes \| \| A and B \| Stress Response \| no subcategory \| Dimethylarginine metabolism \| Ornithine aminotransferase (EC 2.6.1.13) \| yes \| yes \| \| A and B \| Stress Response \| no subcategory \| SigmaB stress responce regulation \| Anti-sigma B factor RsbT \| yes \| yes \| \| A and B \| Stress Response \| no subcategory \| SigmaB stress responce regulation \| Anti-sigma B factor antagonist RsbV \| yes \| yes \| \| A and B \| Stress Response \| no subcategory \| SigmaB stress responce regulation \| RNA polymerase sigma factor SigB \| yes \| yes \| \| A and B \| Stress Response \| no subcategory \| SigmaB stress responce regulation \| RsbS, negative regulator of sigma-B \| yes \| yes \| \| A and B \| Stress Response \| no subcategory \| SigmaB stress responce regulation \| Serine phosphatase RsbU, regulator of sigma subunit \| yes \| yes \| \| A and B \| Stress Response \| no subcategory \| SigmaB stress responce regulation \| Serine-protein kinase RsbW (EC 2.7.11.1) \| yes \| yes \|    |
